# Determination of the Latency Period Between Weekly Gestational Weight Gain and Fetal Growth

**DOI:** 10.1111/ppe.70152

**Published:** 2026-05-10

**Authors:** Madeleine E. St. Ville, Kathryn A. Wagner, Jessica L. Gleason, Katherine L. Grantz, Zhen Chen

**Affiliations:** 1 Biostatistics and Bioinformatics Branch, Division of Population Health Research, Division of Intramural Research, Eunice Kennedy Shriver National Institute of Child Health and Human Development, National Institutes of Health, Bethesda, Maryland, USA; 2 Epidemiology Branch, Division of Population Health Research, Division of Intramural Research, Eunice Kennedy Shriver National Institute of Child Health and Human Development, National Institutes of Health, Bethesda, Maryland, USA

**Keywords:** fetal growth, gestational weight gain, lag time, latency period

## Abstract

**Background:**

Gestational weight gain (GWG) is associated with fetal growth. However, prior studies have assumed specific timing and latency between GWG and fetal growth.

**Objectives:**

We aimed to identify the latency period between the cumulative GWG rate and fetal growth and assess whether accounting for it changes their association.

**Methods:**

We analysed data from the NICHD Fetal Growth Studies–Singletons (*N* = 2445) and NICHD Fetal 3D Study (*n* = 1946). Maternal cumulative GWG rate was the exposure; fetal growth outcomes were estimated fetal weight (EFW) from 2D ultrasound and fractional arm volume (AVol) from 3D. Weekly cumulative GWG rate and fetal growth trajectories were estimated. Two procedures were used to select latency period: one approach identifies the lag with the largest association, and the other the best model fit. Models were adjusted for sociodemographic, clinical, and lifestyle covariates.

**Results:**

Latency selection procedures identified a lag of 7–8 weeks between the cumulative GWG rate and fetal growth. Accounting for this lag, the weekly cumulative GWG rate was positively associated with both EFW and AVol at all gestational weeks evaluated. For example, under the 7-week lag model, for every 1 kg/week increase in the cumulative GWG rate at week 33, there was an average increase of 947 g (95% confidence interval [CI] 894, 1000) in EFW and 7.2 cm^3^ (95% CI 6.6, 7.8) in AVol at week 40. Results were similar under the 8-week lag specification, with overlapping confidence intervals.

**Conclusions:**

Using two common latency selection procedures, we identified a 7–8 week latency period between GWG and subsequent fetal growth, with consistent findings across 2D and 3D fetal growth parameters. These findings highlight the importance of identifying relevant latency periods between GWG and its relationship with fetal growth and the need for improved statistical methods to address the limitations of current latency selection procedures.

## 1 Background

The Institute of Medicine (IOM) has developed gestational weight gain (GWG) recommendations to balance the physiologic needs of pregnancy while minimising health risks for mother and child [1]. However, most individuals gain outside of these recommendations, 21% insufficiently and 47% excessively [2]. Inadequate GWG increases risk of small-for-gestational-age births, while excessive GWG is linked to large-for-gestational-age birthweight and greater adiposity in mothers and offspring [1, 3–5]. It is also associated with altered fetal biometry: inadequate GWG with smaller size [6], and excessive GWG with larger fetal dimensions and weight [6–10].

Prior literature includes several questionable assumptions. First, total GWG assumes constant weight gain throughout pregnancy, limiting assessment of GWG timing relative to fetal growth [6, 10]. In reality, GWG is dynamic: increasing slowly or decreasing in the first trimester then increasing at a relatively constant rate thereafter [1]. This variability, along with the complex dynamics of fetal growth, suggests that the impact of GWG on fetal development may differ across gestation [9]. Second, studies include categorisations such as trimester-specific slopes [7], data-driven clusters [8], or early-, mid-, and late-pregnancy gain [9]. While useful, they may lack sufficient temporal resolution if narrower windows of weight-gain exposure influence fetal growth. By relying on total or coarsely categorised GWG, prior work may obscure important timing-specific effects and fail to capture the true relationship. More precise assessment of GWG timing could therefore improve clinical recommendations and identify optimal intervention windows.

Additionally, when evaluating the relationship between GWG and fetal growth, it is important to account for a latency period—time interval between GWG and its effect on fetal development. Correctly identifying the latency period can help design more effective interventions to address inadequate or excessive GWG. However, prior studies have made differing assumptions on the latency period, while studies evaluating total GWG and fetal growth do not address the temporality of the relationship [6–10]. There is currently no established latency period between GWG and its effects on fetal development. In addition, studies have been limited mainly to investigating the association between GWG and two-dimensional (2D) fetal growth measures and estimated fetal weight (EFW), calculated from 2D fetal biometrics. Newer three-dimensional (3D) measures may provide more insight into the association of GWG with fetal growth by incorporating measures of lean and fat tissue, such as fractional arm volume (AVol) [11]. Therefore, we explored the weekly relationship between GWG and fetal growth using prospective cohort data to directly address key limitations of prior research, including assumptions of constant or broadly categorised GWG, neglect of latency between GWG and fetal growth, and focus on only 2D fetal measures.

## 2 Methods

### 2.1 Study Design and Population

We used data from the Fetal Growth Study—Singletons (2009–2013; *n* = 2802) and from women included in the Fetal 3D Study (2015–2019; *n* = 2675) who had 2-dimensional (2D) and 3D obstetrical ultrasounds collected at 12 US sites [12]. We use the term ‘women’ to retain the original study’s language. The study procedures have been previously described [12]. Briefly, women (18–40, low-risk, singleton pregnancies) were enrolled between 8 weeks (w) 0 days (d) and 13w6d. After an initial sonogram between 11w0d and 13w6d, they were randomised to one of four follow-up schedules, each consisting of up to five sonograms scheduled at prespecified gestational weeks.

Separate analyses were conducted to evaluate the weekly relationship of GWG with EFW and AVol. Each analysis excluded pregnancies that were missing the respective fetal growth parameter measurement, were lost to follow-up or otherwise deactivated from the study, had a non-live birth or missing birth outcomes, had congenital or chromosomal abnormalities, or were missing gestational age at delivery. The resulting sample sizes for the EFW and AVol analyses were 2445 and 1946, respectively (Figures S1 and S2).

### 2.2 Exposure Assessment

At enrollment, women self-reported their pre-pregnancy weight and had their height and weight measured. At each subsequent research visit, maternal weight was measured. Maternal weight was also abstracted from prenatal care records to maximise observations and showed high concordance (*r* = 0.99) with that measured at research visits. Each woman contributed a median of 17 (IQR: 15–19) weight measurements.

### 2.3 Outcome Assessment

At each research ultrasound in the Fetal Growth Studies, 2D fetal measures were obtained, including head circumference (HC), abdominal circumference (AC), and femur length (FL). EFW was calculated using the Hadlock formula [13]. In the Fetal 3D Study, additional fetal measurements were collected by recording and storing fetal volumes during 2D data collection. In both, measurements were taken according to a standardised ultrasound protocol by trained sonographers [12]. Among the available fetal measurements, we selected AVol as an example because of its relatively large sample size across gestation and its ability to quantify soft-tissue volume, which may distinguish between normal and abnormal fetal growth [14]. AVol was based on 50% of the humerus diaphysis length, where the arm sub-volume was subdivided into five equidistant slices that were centred along the mid-humerus. Image slices were circumferentially traced to obtain the measurements [14]. EFW and AVol were available from 15 to 40 weeks’ gestation, with a median of 5 (IQR: 5–6) and 2 (IQR: 2–3) measurements per woman, respectively.

### 2.4 Covariate Assessment

At enrollment, research nurses interviewed women about their sociodemographic characteristics and medical histories. Infant sex was abstracted from the medical record upon delivery. Covariates were selected a priori based on prior literature and included maternal age, pre-pregnancy BMI, race/ethnicity (Asian/Pacific Islander; Hispanic; non-Hispanic Black; non-Hispanic White; interpreted as a social construct), education (less than high school; high school diploma, GED or equivalent; some college or Associate’s degree; Bachelor’s degree; Master’s or Advanced degree), parity (0, 1, and 2 or more), maternal cotinine concentrations, and infant sex.

Pre-pregnancy BMI (kg/m^2^) was calculated from self-reported pre-pregnancy weight and measured height and categorised as normal weight (18.5–24.9 kg/m^2^), overweight (25.0–29.9 kg/m^2^), or obese (≥ 30.0 kg/m^2^). We used the 2009 IOM weight-gain during pregnancy guidelines to categorise total GWG, which was computed as the difference between the self-reported pre-pregnancy weight and the maternal weight at delivery [15]. The IOM guidelines recommend a total gain of 11.3–15.9 kg, 6.8–11.3 kg, and 5.0–9.1 kg for normal, overweight and obesity, respectively [15]. Women then were categorised as having inadequate/adequate/excessive total weight gain if below/within/above guidelines.

### 2.5 Statistical Analyses

Demographic and clinical characteristics of the mothers and their neonates are presented in both the EFW and AVol analyses. Continuous variables are reported as means and standard deviations (SD) and categorical variables as frequencies and relative frequencies.

The analysis was performed in 2 stages. The first separately modelled the individual maternal GWG, EFW, and Avol to obtain weekly trajectories. The second utilised these estimated trajectories to separately model the association between weekly GWG and each fetal growth parameter across various lag specifications, and to use two common latency selection procedures to gain insight into the true latency period.

Specifically, in the first stage, a linear mixed model with cubic splines for the gestational age was used to model maternal weight across gestation, unadjusted for covariates. This modelling approach estimated each individual’s weekly weight trajectory and has the advantage of accounting for differences in the timing and number of weight measurements between women and reducing the influence of measurement error via a smooth weekly trajectory for each individual. The model included fixed effects for the linear, quadratic, and cubic terms, a cubic spline for gestational age, and random effects for the intercept and the gestational age cubic term. The cubic spline included 3 knot points (25th, 50th, and 75th percentiles) at gestational ages. Maternal weight was log-transformed to stabilise variances and improve normality. The weekly weights (kg) were recovered by transforming the estimates back to their original scale. From these trajectory models, we obtained each woman’s weekly cumulative GWG rate (kg/week), calculated as the difference between their weekly estimated maternal weight and pre-pregnancy weight, divided by the number of weeks.

EFW and Avol were modelled separately using the same cubic-spline-based linear mixed model as described above. From these trajectory models, we obtained weekly estimates of each participant’s EFW (g) and AVol (cm^3^) from 15 to 40 weeks of pregnancy.

In the second stage analysis, we fit separate gestational age-specific linear mixed models for EFW and AVol to estimate their separate association with weekly cumulative GWG rate from 15 to 40 weeks of gestation. Both were adjusted for maternal age, pre-pregnancy BMI, race/ethnicity, education, parity, maternal cotinine concentrations, and infant sex. Data were imputed using the regularised iterative factorial analysis for mixed data (FAMD) algorithm for participants with missing covariates (*n* = 128 and 93 for EFW and AVol analysis) [16].

To account for latency, the weekly cumulative GWG rates were lagged by a suitable number of weeks using two latency selection procedures commonly used in the literature. Both procedures fit the model across a range of potential lag times and identify the latency model for statistical inference [17]. One procedure, Effect-Size Lag Approach hereafter, selects the latency period that produces the maximum effect estimates; the other, Best-Fit Lag Approach hereafter, selects the latency period that optimises the model goodness of fit [17]. For both EFW and AVol, we fit the mixed model under lag times from 0 to 14 weeks. Latency periods longer than 14 resulted in degenerated statistical models (i.e., a model matrix that is rank-deficient). For the Effect-Size Lag Approach, we identified the latency model that produced the maximum estimated effects between weekly cumulative GWG rates and the corresponding fetal growth parameter. For the Best-Fit Lag Approach, we chose two commonly used measures of model fit –Akaike Information Criterion (AIC) and Bayesian Information Criterion (BIC) – and identified the latency model that minimised them. Subsequent statistical inference for both EFW and AVol was conducted using the selected lag models. All analyses were performed with R version 4.2.2 [18].

### 2.6 Missing Data

In the AVol analysis, nearly 80% of the excluded pregnancies lacked outcome data (Figure S2). To explore the potential impact of informative missingness, we conducted a sensitivity analysis using inverse probability of censoring weights (IPCW). Stabilised weights were estimated using logistic regression to estimate the probability of a non-missing outcome conditional on baseline covariates. Using these estimated weights, we fit weighted versions of the second-stage linear mixed models under the same range of potential lag times. The selected latency periods and resulting estimates were compared with the unweighted AVol analysis to evaluate the impact of missing data. An IPCW sensitivity analysis was not applied to the EFW analysis because only 5% of the excluded participants were missing EFW outcome measurements (Figure S1), which we deemed negligible and unlikely to have a meaningful impact on the estimation results.

### 2.7 Ethics Approval

Institutional review board approval was obtained by all participating institutions, the data coordinating centre, and the NICHD. Consent was obtained from all participants before data collection.

## 3 Results

The baseline characteristics of the study participants from both analyses are presented in Table 1. In the sample of 2445 women used in the EFW analysis, the mean age was 28.2 years, and 70.3% had at least some college education. This was a diverse sample by study design: 16.0% Asian/Pacific Islander, 28.8% Hispanic, 27.4% non-Hispanic Black, and 27.8% non-Hispanic White. The majority had excessive total GWG (59.6%), while 12.6% had insufficient GWG.

Figure S3 summarises the weekly availability of maternal weight, EFW, and AVol measurements across gestation. All participants contributed a self-reported pre-pregnancy maternal weight. However, maternal weight measurements were relatively sparse early in pregnancy, particularly prior to about week 8 of gestation, and became consistently dense thereafter. EFW and AVol measurements were consistently dense between 15 and 40 weeks of gestation, modestly sparse only at the earlier and later gestational weeks.

**FIGURE 1:**
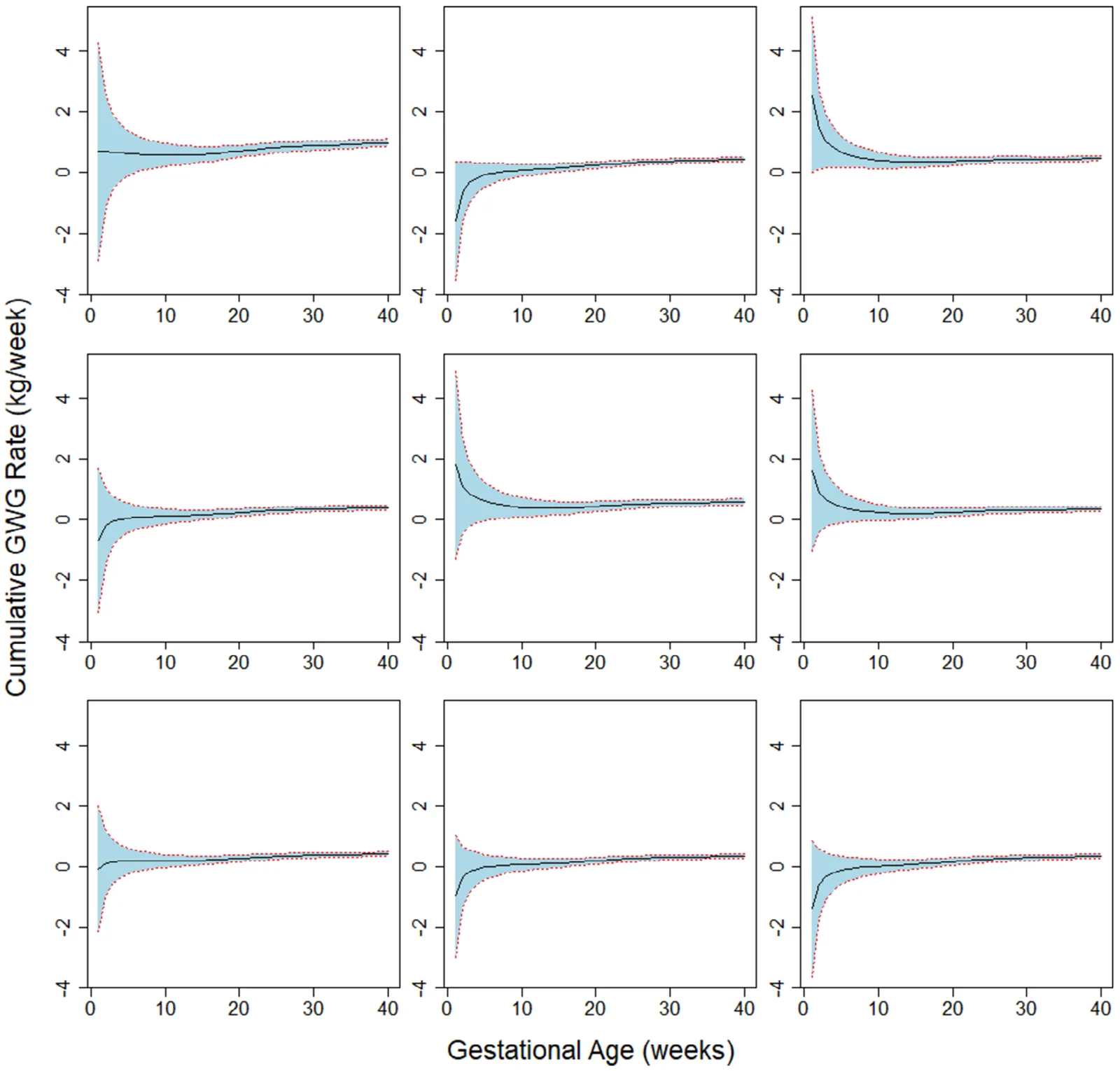
Gestational weight gain trajectories for nine randomly selected study participants; NICHD Fetal Growth Studies—Singletons and the Fetal 3D Study.

**FIGURE 2:**
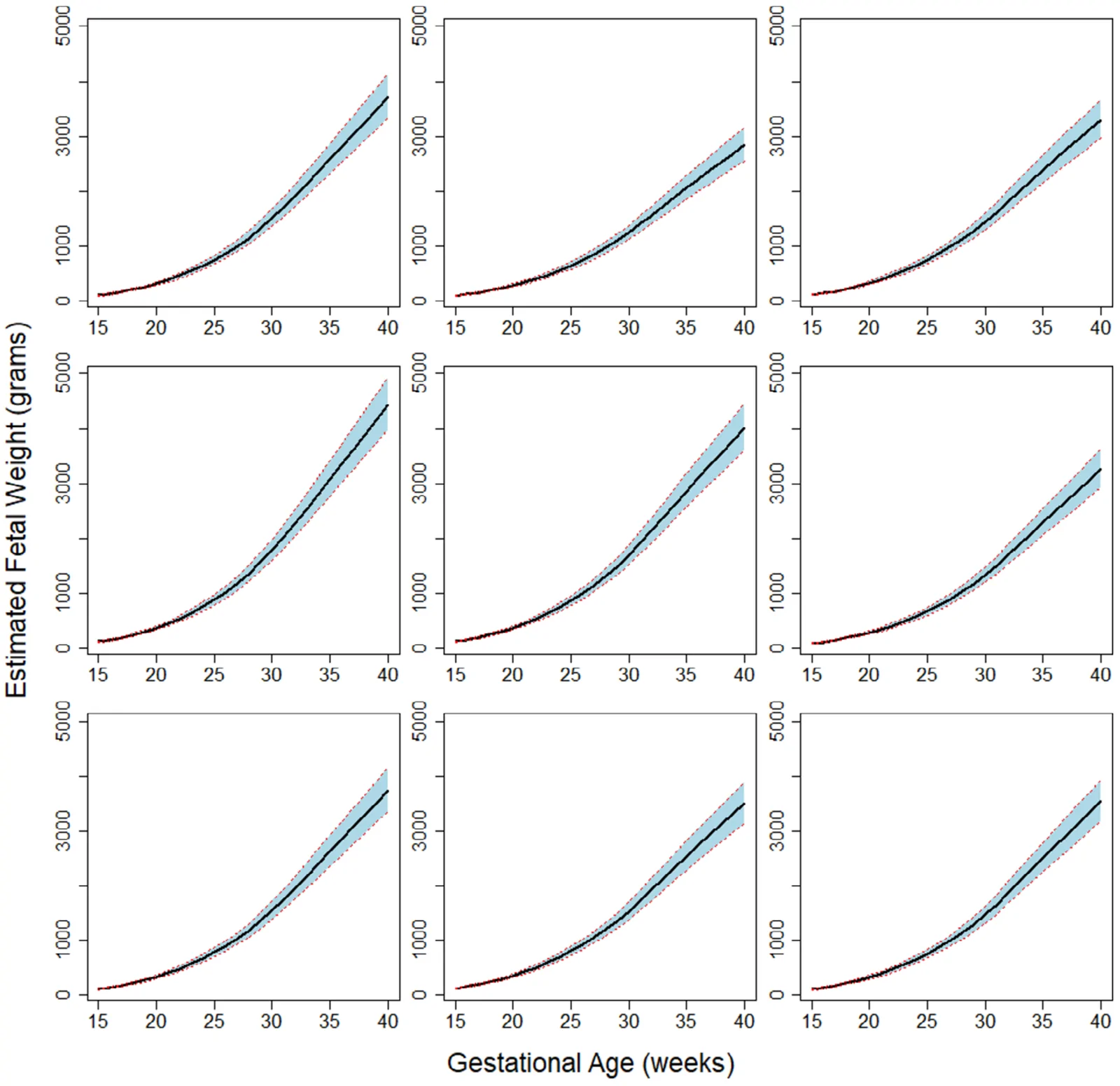
Estimated fetal weight trajectories for nine randomly selected study participants; NICHD Fetal Growth Studies—Singletons and the Fetal 3D Study.

**FIGURE 3:**
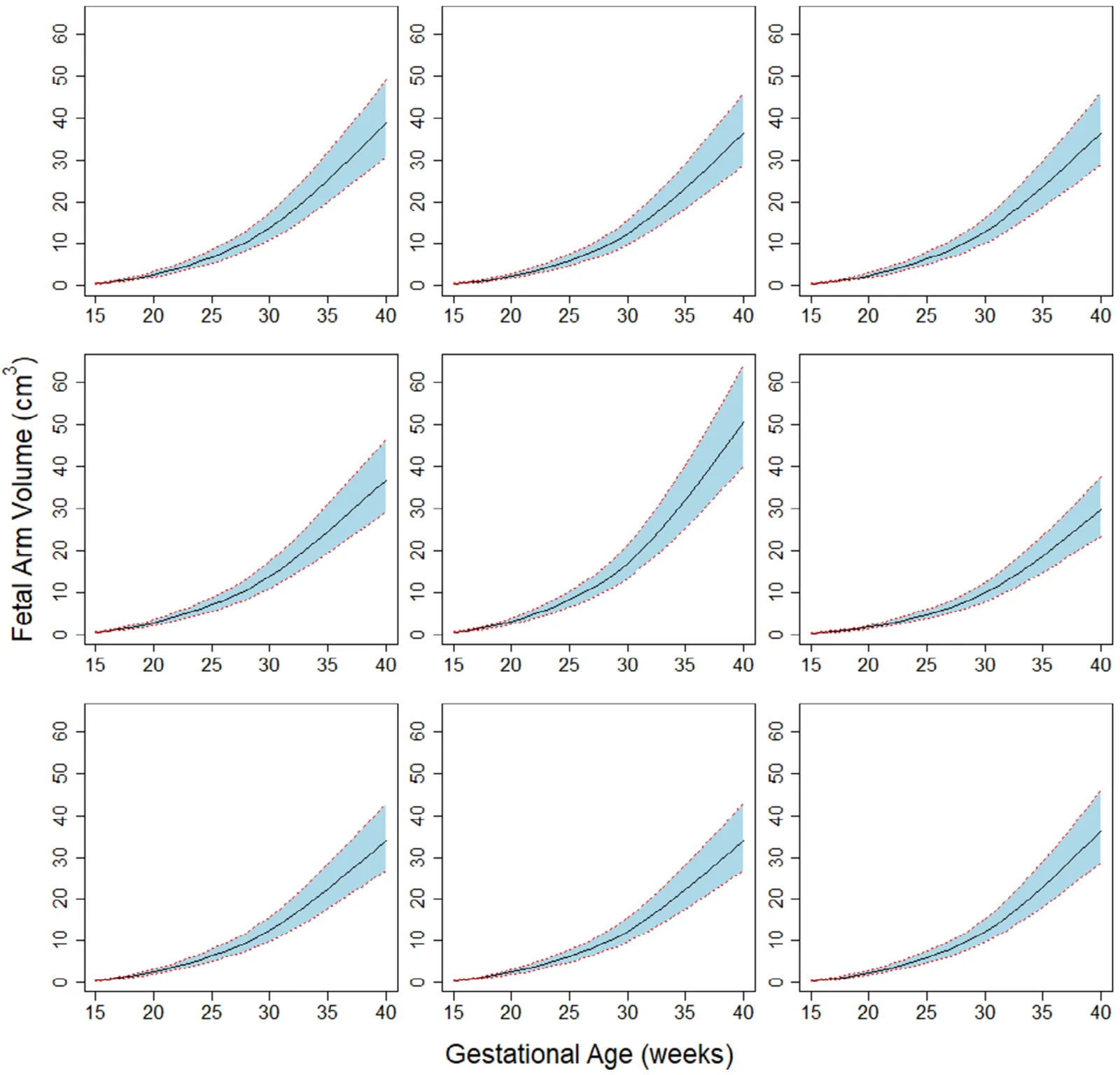
Fractional fetal arm volume trajectories for nine randomly selected study participants; NICHD Fetal Growth Studies—Singletons and the Fetal 3D Study.

Despite these sparsities in the early weeks of gestation, the spline-based linear mixed models used in the first stage analysis leverage information across subjects and time to estimate individual weekly trajectories across gestation, allowing for non-linear growth patterns while accounting for between-subject variability. Estimation results for the fixed-effect spline coefficients and the random-effect variance components from the spline-based mixed models are summarised in Tables S1–S3, and suggest meaningful between-subject variability. Further, the estimated trajectories for nine randomly selected participants (Figures 1–3) demonstrate subject-specific heterogeneity in weekly cumulative GWG rates and fetal growth trajectories, validating the inclusion of random effects in the second-stage models.

The estimation results for the effects of the weekly cumulative GWG rate from the linear mixed models under each lag assumption are reported in Tables S4–S18, and the measures of model fit for each latency model are reported in Table S19. For both EFW and AVol, the 8-week lag model yielded the largest effect estimate at every gestational week (Table S12). Therefore, the Effect-Size Lag Approach selected the model that lagged the weekly cumulative GWG rate by 8 gestational weeks for both EFW and AVol. On the other hand, the 7-week lag model minimised AIC and BIC for EFW and AVol (Table S19). Therefore, under the Best-Fit Lag Approach, the models that lag the weekly cumulative GWG rate by 7 gestational weeks were selected for statistical inference on both EFW and AVol.

**FIGURE 4:**
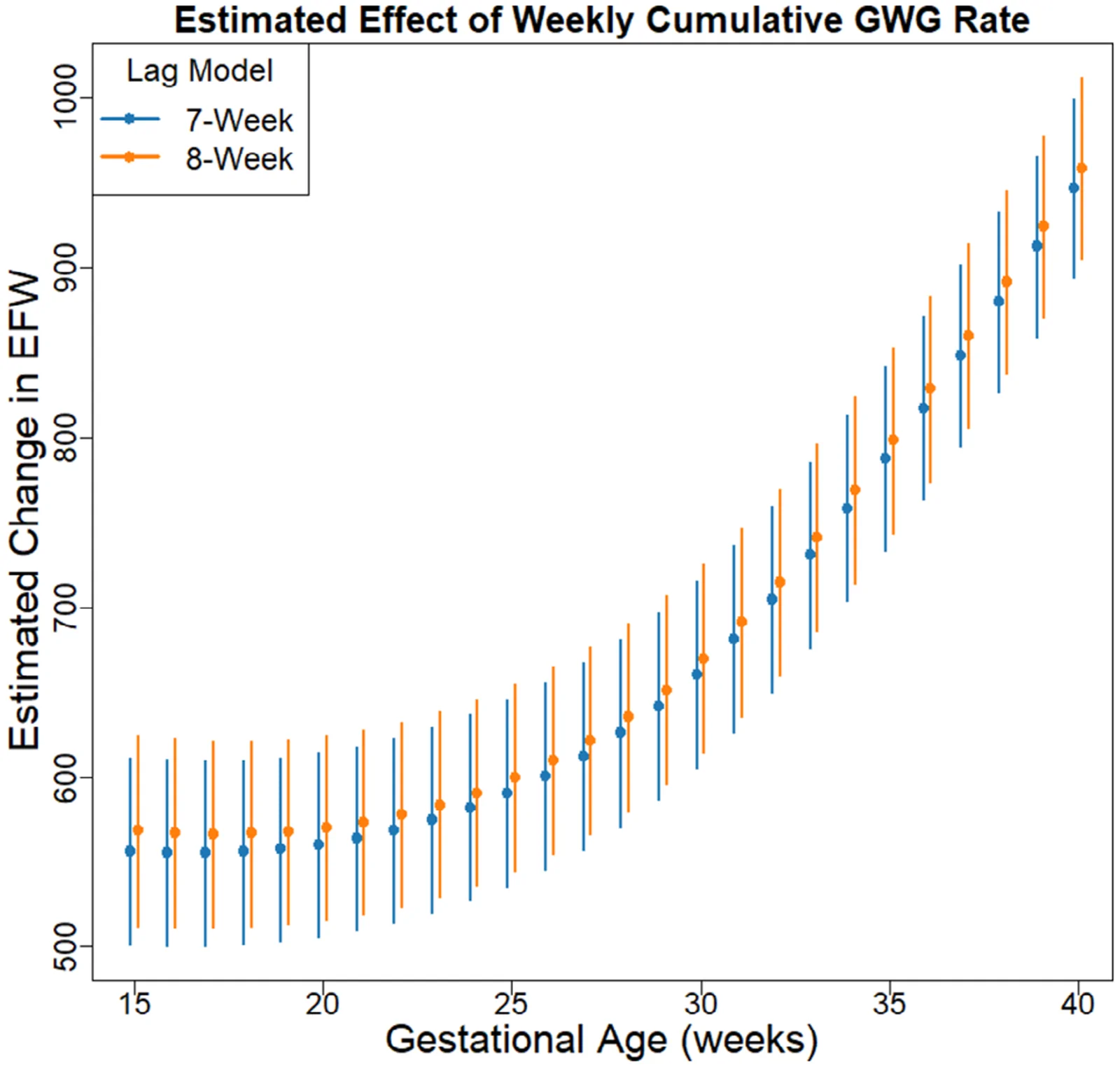
Estimated exposure effect of weekly cumulative gestational weight gain (GWG) rate on estimated fetal weight (EFW), lagged by 7 and 8 weeks; NICHD Fetal Growth Studies—Singletons and the Fetal 3D Study.

Figure 4 displays the estimated effects of the weekly cumulative GWG rate on estimated fetal weight from the two lag models selected from the Effect-Size and Best-Fit Lag approaches. Specifically, the estimated effects of the weekly cumulative GWG rates lagged by 7 weeks are plotted in blue, and those by 8 weeks are plotted in orange. These models account for a 7- and 8-week latency period, respectively, and yield comparable relationship patterns between the weekly cumulative GWG rate and EFW. That is, when the weekly cumulative GWG rate was lagged by either 7 or 8 weeks, it was positively associated with EFW at every gestational week evaluated. For example, when a 7-week latency period between the cumulative GWG rate and its subsequent effect on EFW was assumed, there was a 556 g increase in EFW at 15 weeks (95% CI 501, 511) for every kg/week increase in the cumulative GWG rate at week 8 of pregnancy (Table S11); and by the end of gestation, there was a 947 g (95% CI 894, 1000) increase in EFW at 40 weeks for every kg/week increase in the cumulative GWG rate at week 33. Instead, when an 8-week latency period was assumed, there was a 568 g (95% CI 512, 624) increase in EFW at 15 weeks for every kg/week increase in the cumulative GWG rate at week 7 (Table S12); and by the end of gestation, there was a 959 g (95% CI 905, 1012) increase in EFW at 40 weeks for every kg/week increase in the cumulative GWG rate at week 32 of pregnancy.

**FIGURE 5:**
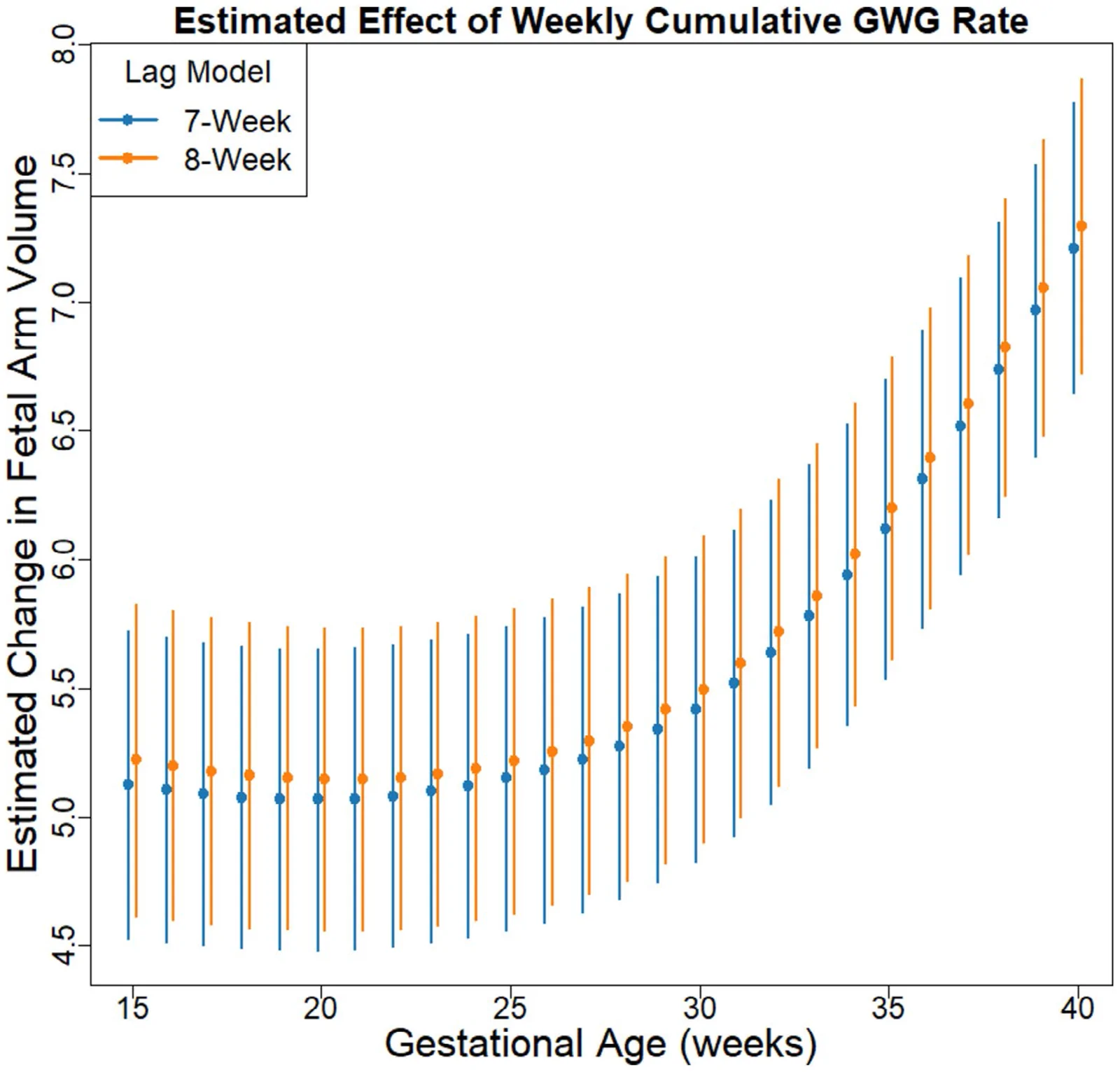
Estimated exposure effect of weekly cumulative gestational weight gain (GWG) rate on fetal fractional arm volume (AVol), lagged by 7 and 8 weeks; NICHD Fetal Growth Studies—Singletons and the Fetal 3D Study.

Similarly, Figure 5 displays the estimated effects of the weekly cumulative GWG rate, lagged by 7 weeks and 8 weeks, on AVol. The two models selected by the latency selection procedures yield similar relationship patterns; i.e., when the weekly cumulative GWG rate is lagged by either 7 weeks or 8 weeks, it is associated with AVol at every gestational week evaluated. For example, when a 7-week latency period between the weekly cumulative GWG rate and its subsequent effect on AVol is assumed, there was a 5.1 cm^3^ (95% CI 4.5, 5.7) increase in AVol at 15 weeks for every kg/week increase in the cumulative GWG rate at week 8 of pregnancy (Table S11); by the end of gestation, there was a 7.2 cm^3^ (95% CI 6.6, 7.8)increase in AVol at 40 weeks for every kg/week increase in the cumulative GWG rate at week 33. On the other hand, when an 8-week latency period is assumed, there was a 5.2 cm^3^ (95% CI 4.6, 5.8) increase in AVol at 15 weeks for every kg/week increase in the cumulative GWG rate at week 7 (Table S12); by the end of gestation, there was a 7.3 cm^3^ (95% CI 6.7, 7.9) increase in AVol at 40 weeks for every kg/week increase in the cumulative GWG rate at week 32 (Table S12).

Results from the IPCW sensitivity analysis for the AVol analysis were consistent with the primary analysis. The same 7-week and 8-week lag periods were identified by the latency selection procedures, and the corresponding effect estimates were similar, suggesting that the patterns observed in the primary AVol analysis were robust to excluding cases with missing outcome data (see Tables S20–S35).

## 4 Comment

### 4.1 Principal Findings

Using two common latency selection procedures, we identified a 7- to 8-week lag between the weekly cumulative GWG rate and fetal growth from 15 to 40 weeks’ gestation. The Effect-Size Lag Approach selected an 8-week latency period, while the Best-Fit Lag Approach selected a 7-week latency period for both EFW and AVol. Lagged weekly GWG was associated with increases in EFW and AVol at each gestational week, with effect estimates from both latency models producing overlapping confidence intervals. These findings suggest that a latency period exists between weekly GWG and fetal growth, though the exact duration may differ depending on the selection procedure. Our findings are novel, as previous studies have assumed no lag time between GWG and subsequent fetal growth or have used total GWG assessed at delivery, and thus cannot temporally precede fetal growth [6–10].

### 4.2 Strengths of the Study

This study overcomes several common limitations of prior work. We used weekly cumulative GWG, thereby avoiding assumptions of constant GWG across pregnancy or reliance on broad interval measures. Additionally, we specifically address the lack of biologically informed latency modelling by estimating the lag between the weekly cumulative GWG rate and fetal growth via common latency selection procedures. Two fetal growth outcomes were considered: EFW derived from 2D fetal biometric measurements and fractional arm volume (AVol), a 3D soft-tissue measure that captures lean and fat tissue accumulation. Weekly maternal weight trajectories and fetal growth trajectories were estimated using spline-based linear mixed models, allowing for non-linear patterns and subject-specific variability. Sensitivity analyses using inverse probability of censoring weights (IPCW) indicated that our findings are robust to excluding cases with missing outcome data.

### 4.3 Limitations of the Data

First, early gestational associations relied on trajectory-based estimates derived from relatively sparse early-pregnancy weight measurements and self-reported pre-pregnancy weight, which may introduce additional uncertainty. Second, the spline-based models used to estimate the weekly trajectories may not fully capture the non-linear variability in maternal weight gain or fetal growth. Third, both latency selection procedures assume a fixed latency period across gestation and participants, which may oversimplify biological variability. Fourth, the prespecified range of potential lag times could limit the detection of biologically relevant latency periods beyond 0–14 weeks. The Effect-Size Lag Approach could produce biased estimates, particularly when the true lag time falls outside the pre-specified range of exposure lag assumptions, and the Best-Fit Lag Approach could underestimate variability and yield confidence intervals that are too narrow [17]. The Best-Fit Lag Approach assumes the selected model is true, estimating variance from a single model and hence ignoring model uncertainty [17].

These limitations highlight areas for future methodological development. Work could focus on developing a modelling technique that simultaneously estimates weekly effects and individualised or time-varying latency periods. Distributed lag modelling frameworks incorporating a weighted rather than an unweighted cumulative GWG could more accurately reflect the timing and intensity of maternal weight gain. Additionally, methods integrating uncertainty in latency selection or comparing multiple lagged models could further improve statistical efficiency, biological interpretability, and generalisability across populations.

### 4.4 Interpretation

The observed 7–8 week latency period for the cumulative GWG rate indicates that shorter assumed lag times in prior studies may have masked the true association between GWG and fetal growth. Moreover, although our findings suggest similar lag times for EFW and AVol, the latency period may vary depending on the fetal growth parameter assessed. Considering evidence of organ sparing and differential accumulation of fat mass among foetuses exposed to non-optimal maternal nutritional environments [19, 20], it is likely that latency periods may differ across fetal 3D structures. Identification of appropriate latency periods can improve study design by informing the timing of data collection, improve statistical efficiency, and enhance the biological interpretability of weekly GWG effects. Conflicting latency results across the two selection procedures highlight the importance of carefully evaluating method choice and demonstrate that widely used latency approaches have inherent limitations that may affect model estimates. Finally, understanding latency periods may inform the timing of interventions and updates to clinical GWG recommendations, providing insight into biological windows of fetal plasticity.

## 5 Conclusions

Weekly cumulative GWG rate is positively associated with subsequent fetal growth after a 7- to 8-week latency period, with consistent patterns across both 2D and 3D fetal growth parameters. Although these preliminary results should be replicated in other populations and with additional fetal growth parameters, these findings highlight the importance of evaluating temporally lagged associations between maternal weight gain and fetal growth. Future work should focus on developing advanced modelling approaches, including distributed lag models, individualised or time-varying latency estimation, and methods that incorporate the uncertainty in latency selection, to address remaining limitations in latency modelling.

## Supplementary Material

supp

Additional supporting information can be found online in the Supporting Information section. **Figure S1:** Flowchart for inclusion of study participants for the estimated fetal weight (EFW) analysis in the NICHD Fetal Growth Studies—Singletons. **Figure S2:** Flowchart for inclusion of study participants for the fractional arm volume (AVol) analysis in the NICHD Fetal Growth Studies—Singletons and the Fetal 3D Study. **Figure S3:** Weekly availability of maternal weight and fetal ultrasound data across gestation. Line plots display the number of maternal weight measurements and ultrasound examinations contributing to estimated fetal weight (EFW) and fractional arm volume (AVol) trajectories at each gestational week; NICHD Fetal Growth Studies—Singletons and Fetal 3D Study. **Table S1:** Estimation results for the fixed effects and random effects variance components from the spline-based linear mixed model used to estimate the individual maternal weight trajectories; NICHD Fetal Growth Studies—Singletons and Fetal 3D Study. **Table S2:** Estimation results for the fixed effects and random effects variance components from the spline-based linear mixed model used to estimate the individual estimated fetal weight (EFW) trajectories; NICHD Fetal Growth Studies—Singletons and Fetal 3D Study. **Table S3:** Estimation results for the fixed effects and random effects variance components from the spline-based linear mixed model used to estimate the individual fractional fetal arm volume (AVol) trajectories; NICHD Fetal Growth Studies—Singletons and Fetal 3D Study. **Table S4:** Weekly effect estimates under the 0-week lag models; NICHD Fetal Growth Studies—Singletons and Fetal 3D Study. **Table S5:** Weekly effect estimates under the 1-week lag models; NICHD Fetal Growth Studies—Singletons and Fetal 3D Study. **Table S6:** Weekly effect estimates under the 2-week lag models; NICHD Fetal Growth Studies—Singletons and Fetal 3D Study. **Table S7:** Weekly effect estimates under the 3-week lag models; NICHD Fetal Growth Studies—Singletons and Fetal 3D Study. **Table S8:** Weekly effect estimates under the 4-week lag models; NICHD Fetal Growth Studies—Singletons and Fetal 3D Study. **Table S9:** Weekly effect estimates under the 5-week lag models; NICHD Fetal Growth Studies—Singletons and Fetal 3D Study. **Table S10:** Weekly effect estimates under the 6-week lag models; NICHD Fetal Growth Studies—Singletons and Fetal 3D Study. **Table S11:** Weekly effect estimates under the 7-week lag models; NICHD Fetal Growth Studies—Singletons and Fetal 3D Study. **Table S12:** Weekly effect estimates under the 8-week lag models; NICHD Fetal Growth Studies—Singletons and Fetal 3D Study. **Table S13:** Weekly effect estimates under the 9-week lag models; NICHD Fetal Growth Studies—Singletons and Fetal 3D Study. **Table S14:** Weekly effect estimates under the 10-week lag models; NICHD Fetal Growth Studies—Singletons and Fetal 3D Study. **Table S15:** Weekly effect estimates under the 11-week lag models; NICHD Fetal Growth Studies—Singletons and Fetal 3D Study. **Table S16:** Weekly effect estimates under the 12-week lag models; NICHD Fetal Growth Studies—Singletons and Fetal 3D Study. **Table S17:** Weekly effect estimates under the 13-week lag models; NICHD Fetal Growth Studies—Singletons and Fetal 3D Study. **Table S18:** Weekly effect estimates under the 14-week lag models; NICHD Fetal Growth Studies—Singletons and Fetal 3D Study. **Table S19:** Measures of model fit under each of the lag specifications for estimated fetal weight and fractional fetal arm volume; NICHD Fetal 3D Study—Singletons, 2015–2019; NICHD Fetal Growth Studies—Singletons and Fetal 3D Study. **Table S20:** Measures of model fit under each of the lag specifications for fractional fetal arm volume from IPCW sensitivity analysis; NICHD Fetal 3D Study—Singletons, 2015–2019; NICHD Fetal Growth Studies—Singletons and Fetal 3D Study. **Table S21:** Weekly effect estimates under the 0-week lag model for fractional fetal arm volume from IPCW sensitivity analysis; NICHD Fetal Growth Studies—Singletons and Fetal 3D Study. **Table S22:** Weekly effect estimates under the 1-week lag model for fractional fetal arm volume from IPCW sensitivity analysis; NICHD Fetal Growth Studies—Singletons and Fetal 3D Study. **Table S23:** Weekly effect estimates under the 2-week lag model for fractional fetal arm volume from IPCW sensitivity analysis; NICHD Fetal Growth Studies—Singletons and Fetal 3D Study. **Table S24:** Weekly effect estimates under the 3- week lag model for fractional fetal arm volume from IPCW sensitivity analysis; NICHD Fetal Growth Studies—Singletons and Fetal 3D Study. **Table S25:** Weekly effect estimates under the 4-week lag model for fractional fetal arm volume from IPCW sensitivity analysis; NICHD Fetal Growth Studies—Singletons and Fetal 3D Study. **Table S26:** Weekly effect estimates under the 5-week lag model for fractional fetal arm volume from IPCW sensitivity analysis; NICHD Fetal Growth Studies—Singletons and Fetal 3D Study. **Table S27:** Weekly effect estimates under the 6-week lag model for fractional fetal arm volume from IPCW sensitivity analysis; NICHD Fetal Growth Studies—Singletons and Fetal 3D Study. **Table S28:** Weekly effect estimates under the 7-week lag model for fractional fetal arm volume from IPCW sensitivity analysis; NICHD Fetal Growth Studies—Singletons and Fetal 3D Study. **Table S29:** Weekly effect estimates under the 8-week lag model for fractional fetal arm volume from IPCW sensitivity analysis; NICHD Fetal Growth Studies—Singletons and Fetal 3D Study. **Table S30:** Weekly effect estimates under the 9-week lag model for fractional fetal arm volume from IPCW sensitivity analysis; NICHD Fetal Growth Studies—Singletons and Fetal 3D Study. **Table S31:** Weekly effect estimates under the 10-week lag model for fractional fetal arm volume from IPCW sensitivity analysis; NICHD Fetal Growth Studies—Singletons and Fetal 3D Study. **Table S32:** Weekly effect estimates under the 11-week lag model for fractional fetal arm volume from IPCW sensitivity analysis; NICHD Fetal Growth Studies—Singletons and Fetal 3D Study. **Table S33:** Weekly effect estimates under the 12-week lag model for fractional fetal arm volume from IPCW sensitivity analysis; NICHD Fetal Growth Studies—Singletons and Fetal 3D Study. **Table S34:** Weekly effect estimates under the 13-week lag model for fractional fetal arm volume from IPCW sensitivity analysis; NICHD Fetal Growth Studies—Singletons and Fetal 3D Study. **Table S35:** Weekly effect estimates under the 14- week lag model for fractional fetal arm volume from IPCW sensitivity analysis; NICHD Fetal Growth Studies—Singletons and Fetal 3D Study.

## Figures and Tables

**TABLE 1:** Demographic and medical characteristics of study samples; NICHD Fetal 3D Study—Singletons, 2015–2019.

	Estimated fetal weight (*n* = 2445)	Fractional fetal arm volume (*n* = 1946)
	Mean (SD) or *n* (%)	Mean (SD) or *n* (%)
Maternal age, years	28.2 (5.5)	28.3 (5.4)
Pre-pregnancy BMI, kg/m^2^	25.4 (5.1)	25.1 (5.0)
Pre-pregnancy BMI		
Normal weight	1364 (55.8)	1142 (58.7)
Overweight	668 (27.3)	510 (26.2)
Obese	413 (16.9)	294 (15.1)
Total Gestational Weight Gain based on 2009 IOM Guidelines		
Inadequate	307 (12.6)	234 (12.0)
Adequate	680 (27.8)	550 (28.3)
Excessive	1458 (59.6)	1162 (59.7)
Maternal race/ethnicity		
Asian/Pacific Islander	392 (16.0)	303 (15.6)
Hispanic	703 (28.8)	483 (24.8)
Non-Hispanic Black	670 (27.4)	548 (28.2)
Non-Hispanic White	680 (27.8)	612 (31.4)
Maternal education		
Less than high school	280 (11.5)	184 (9.5)
High school diploma, GED, or equivalent	447 (18.3)	328 (16.9)
Some college or Associate’s degree	728 (29.8)	578 (29.7)
Bachelor’s degree	582 (23.8)	492 (25.3)
Master’s or Advanced degree	408 (16.7)	364 (18.7)
Cotinine, ng/mL	1.5 (13.8)	1.4 (13.9)
Parity		
0	1142 (46.7)	932 (47.9)
1	837 (34.2)	669 (34.4)
2 or more	466 (19.1)	345 (17.7)
Infant sex		
Male	1248 (51.0)	1008 (51.8)
Female	1197 (49.0)	938 (48.2)

Abbreviations: BMI, body mass index; GED, general educational diploma; IOM, Institute of Medicine.

## Data Availability

The data, along with a set of guidelines for researchers applying for access, will be posted to the NICHD/DIPHR Biospecimen Repository Access and Data Sharing [https://brics.cit.nih.gov/] (BRICS).

## References

[1] Rasmussen KM and Yaktine AL, Weight Gain During Pregnancy (National Academies Press (US), 2009). 20669500

[2] Deputy NP, Sharma AJ, Kim SY, and Hinkle SN, “Prevalence and Characteristics Associated With Gestational Weight Gain Adequacy,” Obstetrics and Gynecology 125, no. 4 (2015): 773–781. 25751216 10.1097/AOG.0000000000000739 PMC4425284

[3] Pugh SJ, Albert PS, Kim S, , “Patterns of Gestational Weight Gain and Birthweight Outcomes in the Eunice Kennedy Shriver National Institute of Child Health and Human Development Fetal Growth Studies-Singletons: A Prospective Study,” American Journal of Obstetrics and Gynecology 217, no. 3 (2017): 346.e11–346.e11. 10.1016/j.ajog.2017.05.013 PMC5581247 28502760

[4] Goldstein RF, Abell SK, Ranasinha S, , “Association of Gestational Weight Gain With Maternal and Infant Outcomes: A Systematic Review and Meta-Analysis,” Journal of the American Medical Association 317, no. 21 (2017): 2207–2225. 28586887 10.1001/jama.2017.3635 PMC5815056

[5] Siega-Riz AM, Viswanathan M, Moos MK, , “A Systematic Review of Outcomes of Maternal Weight Gain According to the Institute of Medicine Recommendations: Birthweight, Fetal Growth, and Postpartum Weight Retention,” American Journal of Obstetrics and Gynecology 201, no. 4 (2009): 339.e1–339.e14. 10.1016/j.ajog.2009.07.002 19788965

[6] Hure AJ, Collins CE, Giles WB, Paul JW, and Smith R, “Greater Maternal Weight Gain During Pregnancy Predicts a Large but Lean Fetal Phenotype: A Prospective Cohort Study,” Maternal and Child Health Journal 16, no. 7 (2012): 1374–1384. 22052171 10.1007/s10995-011-0904-8

[7] Elhddad AS, Fairlie F, and Lashen H, “Impact of Gestational Weight Gain on Fetal Growth in Obese Normoglycemic Mothers: A Comparative Study,” Acta Obstetricia et Gynecologica Scandinavica 93, no. 8 (2014): 771–777. 24832777 10.1111/aogs.12427

[8] Galjaard S, Pexsters A, Devlieger R, , “The Influence of Weight Gain Patterns in Pregnancy on Fetal Growth Using Cluster Analysis in an Obese and Nonobese Population,” Obesity (Silver Spring) 21, no. 7 (2013): 1416–1422. 23408453 10.1002/oby.20348

[9] Hinkle SN, Johns AM, Albert PS, Kim S, and Grantz KL, “Longitudinal Changes in Gestational Weight Gain and the Association With Intrauterine Fetal Growth,” European Journal of Obstetrics Gynecology and Reproductive Biology 190 (2015): 41–47. 25978857 10.1016/j.ejogrb.2015.04.006 PMC4458401

[10] Young MF, Hong Nguyen P, Addo OY, , “Timing of Gestational Weight Gain on Fetal Growth and Infant Size at Birth in Vietnam,” PLoS One 12, no. 1 (2017): e0170192. 28114316 10.1371/journal.pone.0170192 PMC5256875

[11] Grantz KL, Lee W, Mack LM, , “Multiethnic Growth Standards for Fetal Body Composition and Organ Volumes Derived From 3D Ultrasonography,” American Journal of Obstetrics and Gynecology 232, no. 3 (2024): 324.e1–324.e160. 10.1016/j.ajog.2024.05.049 PMC11612034 38838912

[12] Buck Louis GM, Grewal J, Albert PS, , “Racial/Ethnic Standards for Fetal Growth, the NICHD Fetal Growth Studies,” American Journal of Obstetrics and Gynecology 213, no. 4 (2015): 449.e441. 10.1016/j.ajog.2015.08.032 PMC4584427 26410205

[13] Hadlock F, Harrist R, Sharman R, Deter R, and Park S, “Estimation of Fetal Weight With the Use of Head, Body, and Femur Measurements - a Prospective Study,” American Journal of Obstetrics and Gynecology 151, no. 3 (1985): 333–337. 3881966 10.1016/0002-9378(85)90298-4

[14] Grantz KL, Lee W, Chen Z, , “The NICHD Fetal 3D Study: A Pregnancy Cohort Study of Fetal Body Composition and Volumes,” American Journal of Epidemiology 193, no. 4 (2024): 580–595. 37946325 10.1093/aje/kwad210 PMC11484591

[15] American College of Obstetrics and Gynecology, “ACOG Committee Opinion No. 548: Weight Gain During Pregnancy,” Obstetrics and Gynecology 121 (2013): 210–212. 23262962 10.1097/01.aog.0000425668.87506.4c

[16] Audigier V, Husson F, and Josse J, “A Principle Component Method to Impute Missing Values for Mixed Data,” Advances in Data Analysis and Classification 10 (2014): 5–26.

[17] Richardson DB, Cole SR, Chu H, and Langholz B, “Lagging Exposure Information in Cumulative Exposure-Response Analysis,” American Journal of Epidemiology 174, no. 12 (2011): 1416–1422. 22047823 10.1093/aje/kwr260 PMC3276301

[18] R Core Team, R: A Language and Environment for Statistical Computing (R Foundation for Statistical Computing, 2022).

[19] Catalano PM, Presley L, Minium J, and Hauguel-de Mouzon S, “Fetuses of Obese Mothers Develop Insulin Resistance In Utero,” Diabetes Care 32, no. 6 (2009): 1076–1080. 19460915 10.2337/dc08-2077 PMC2681036

[20] Serpente P, Zhang Y, Islimye E, Hart-Johnson S, and Gould AP, “Quantification of Fetal Organ Sparing in Maternal Low-Protein Dietary Models,” Wellcome Open Research 6 (2022): 218. 35634534 10.12688/wellcomeopenres.17124.1 PMC9120932

