# Supplementary material for: Determination of the Latency Period Between Weekly Gestational Weight Gain and Fetal Growth
Source: Paediatr Perinat Epidemiol. Author manuscript; Available in PMC 2026 Jun 23. (PMC13288335; doi:10.1111/ppe.70152)
Supplement: supp [file NIHMS2177023-supplement-supp.docx]

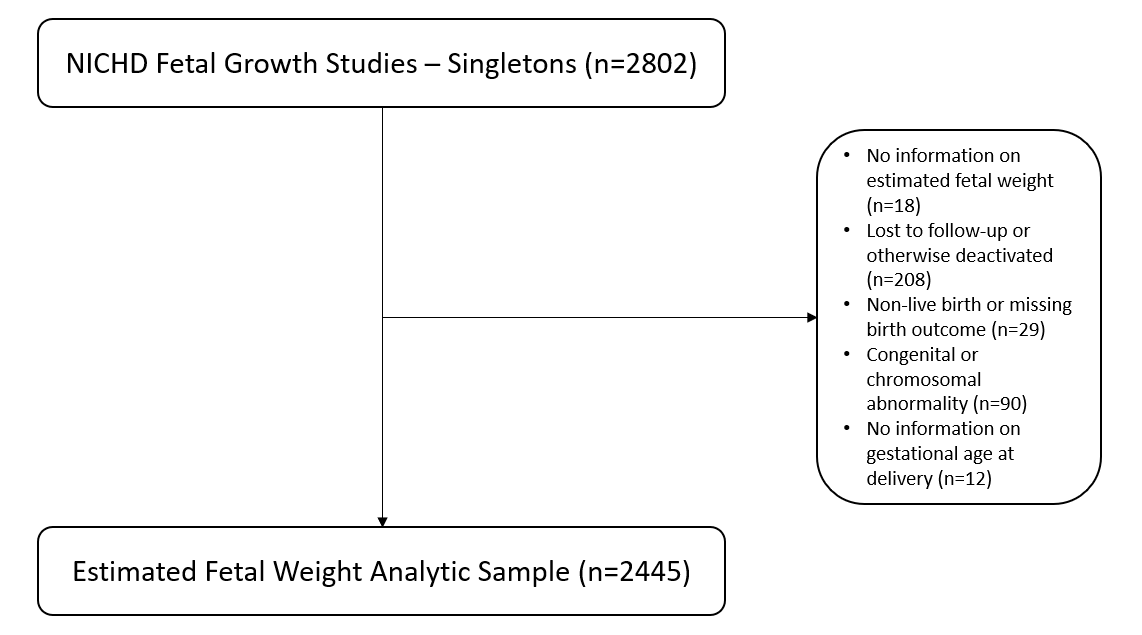


**Figure S1.** Flowchart for inclusion of study participants for the estimated fetal weight (EFW) analysis in the NICHD Fetal Growth Studies – Singletons.


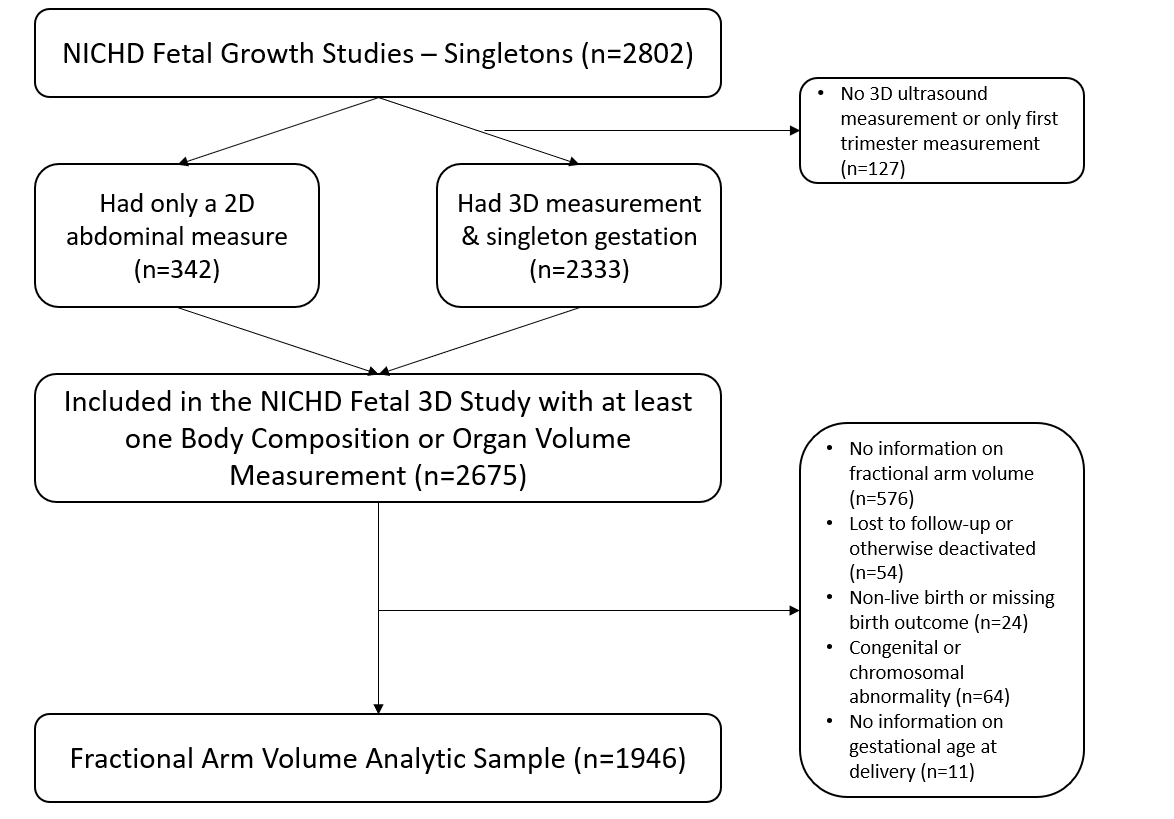


**Figure S2.** Flowchart for inclusion of study participants for the fractional arm volume (AVol) analysis in the NICHD Fetal Growth Studies – Singletons and the Fetal 3D Study.


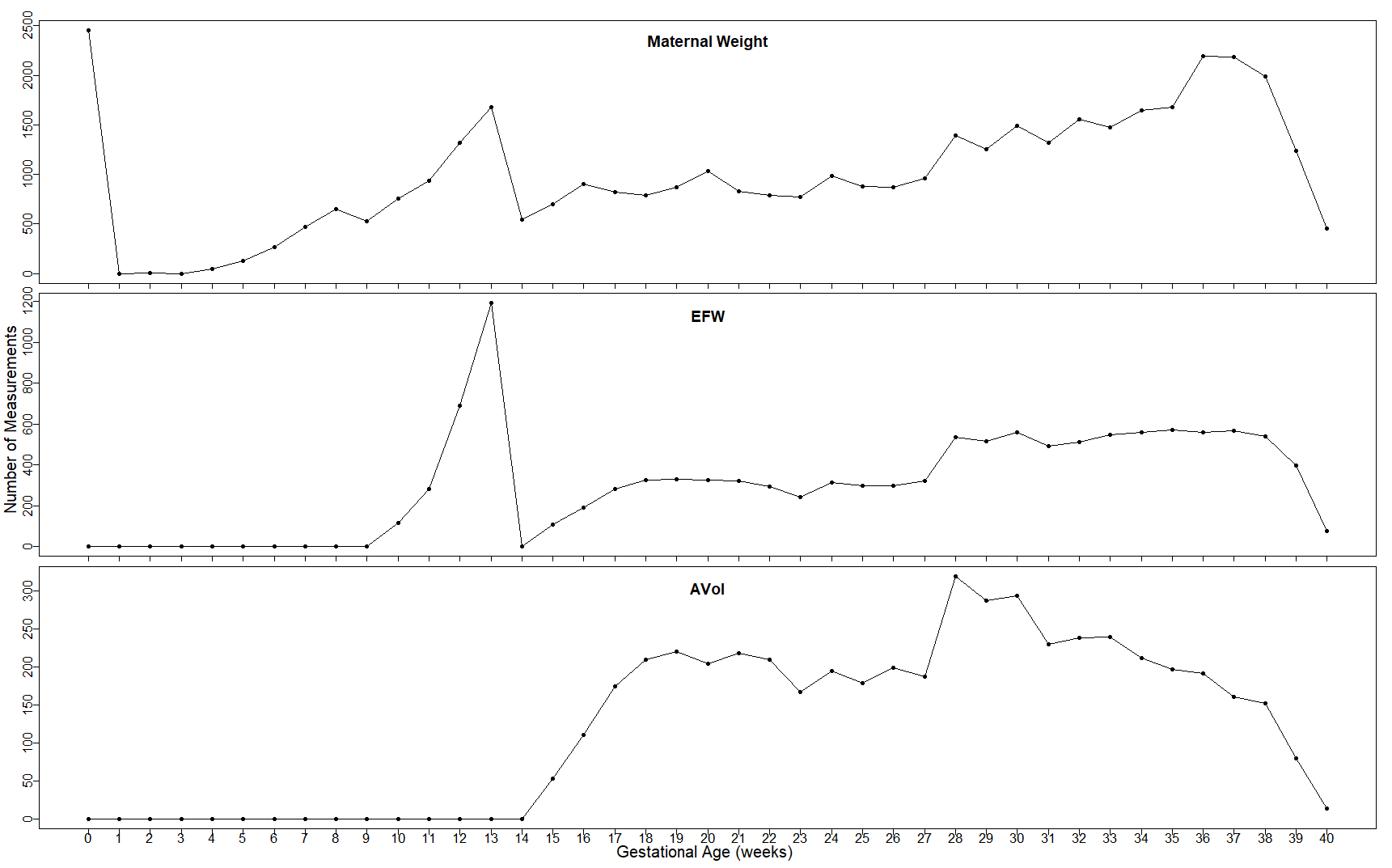


**Figure S3.** Weekly availability of maternal weight and fetal ultrasound data across gestation. Line plots display the number of maternal weight measurements and ultrasound examinations contributing to estimated fetal weight (EFW) and fractional arm volume (AVol) trajectories at each gestational week; NICHD Fetal Growth Studies – Singletons and Fetal 3D Study.

^a^ Note that the EFW and AVol measurements prior to week 15 were excluded from the first- and second-stage models of the study.

**Table S1.** Estimation results for the fixed effects and random effects variance components from the spline-based linear mixed model used to estimate the individual maternal weight trajectories; NICHD Fetal Growth Studies - Singletons and Fetal 3D Study.

| Model Parameter | Estimate | Standard Error |
| --- | --- | --- |
| ***Fixed Effects*** |  |  |
| GA | 0.005 | 0.0002 |
| GA^2^ | -0.0005 | 0.00002 |
| GA^3^ | 0.00002 | 0.0000007 |
| ***Variance Components*** |  |  |
| Random Intercept | 0.049 | 0.221 |
| Random Slope | 0.000005 | 0.002 |
| Residuals | 0.0004 | 0.020 |

GA: Gestational Age

**Table S2.** Estimation results for the fixed effects and random effects variance components from the spline-based linear mixed model used to estimate the individual estimated fetal weight (EFW) trajectories; NICHD Fetal Growth Studies - Singletons and Fetal 3D Study.

| Model Parameter | Estimate | Standard Error |
| --- | --- | --- |
| ***Fixed Effects*** |  |  |
| GA | 0.007 | 0.023 |
| GA^2^ | 0.017 | 0.001 |
| GA^3^ | -0.0004 | 0.00003 |
| ***Variance Components*** |  |  |
| Random Intercept | 0.022 | 0.147 |
| Random Slope | 0.00003 | 0.005 |
| Residuals | 0.003 | 0.054 |

GA: Gestational Age

**Table S3.** Estimation results for the fixed effects and random effects variance components from the spline-based linear mixed model used to estimate the individual fractional fetal arm volume (AVol) trajectories; NICHD Fetal Growth Studies - Singletons and Fetal 3D Study.

| Model Parameter | Estimate | Standard Error |
| --- | --- | --- |
| ***Fixed Effects*** |  |  |
| GA | 1.576 | 0.250 |
| GA^2^ | -0.053 | 0.012 |
| GA^3^ | 0.0007 | 0.0002 |
| ***Variance Components*** |  |  |
| Random Intercept | 0.094 | 0.307 |
| Random Slope | 0.00009 | 0.010 |
| Residuals | 0.014 | 0.119 |

GA: Gestational Age

| **Table S4.** Weekly effect estimates under the 0-week lag models; NICHD Fetal Growth Studies - Singletons and Fetal 3D Study. | | | | | | |
| --- | --- | --- | --- | --- | --- | --- |
|  | Estimated Fetal Weight (EFW) | | | Fractional Fetal Arm Volume (AVol) | | |
| Gestational Age, week | Estimate | Standard Error | 95% Confidence Interval | Estimate | Standard Error | 95% Confidence Interval |
| 15 | 390.75 | 27.32 | (337.20, 444.31) | 3.73 | 0.30 | (3.15, 4.31) |
| 16 | 391.56 | 27.43 | (337.81, 445.32) | 3.72 | 0.30 | (3.14, 4.31) |
| 17 | 392.58 | 27.55 | (338.58, 446.58) | 3.72 | 0.30 | (3.13, 4.30) |
| 18 | 393.82 | 27.67 | (339.58, 448.06) | 3.71 | 0.30 | (3.12, 4.30) |
| 19 | 395.35 | 27.78 | (340.89, 449.80) | 3.70 | 0.30 | (3.11, 4.29) |
| 20 | 397.29 | 27.86 | (342.68, 451.91) | 3.69 | 0.30 | (3.10, 4.28) |
| 21 | 399.86 | 27.91 | (345.15, 454.57) | 3.69 | 0.30 | (3.10, 4.28) |
| 22 | 403.28 | 27.93 | (348.54, 458.03) | 3.69 | 0.30 | (3.10, 4.28) |
| 23 | 407.82 | 27.92 | (353.11, 462.54) | 3.69 | 0.30 | (3.10, 4.28) |
| 24 | 413.74 | 27.87 | (359.12, 468.37) | 3.70 | 0.30 | (3.11, 4.29) |
| 25 | 421.31 | 27.80 | (366.83, 475.80) | 3.72 | 0.30 | (3.13, 4.31) |
| 26 | 430.79 | 27.70 | (376.49, 485.09) | 3.75 | 0.30 | (3.17, 4.34) |
| 27 | 442.40 | 27.59 | (388.31, 496.48) | 3.79 | 0.30 | (3.21, 4.38) |
| 28 | 456.35 | 27.48 | (402.50, 510.20) | 3.85 | 0.30 | (3.27, 4.43) |
| 29 | 472.80 | 27.35 | (419.19, 526.41) | 3.92 | 0.29 | (3.35, 4.50) |
| 30 | 491.78 | 27.22 | (438.42, 545.13) | 4.01 | 0.29 | (3.44, 4.59) |
| 31 | 513.21 | 27.09 | (460.11, 566.31) | 4.12 | 0.29 | (3.55, 4.69) |
| 32 | 536.96 | 26.96 | (484.12, 589.80) | 4.25 | 0.29 | (3.68, 4.82) |
| 33 | 562.81 | 26.82 | (510.24, 615.39) | 4.39 | 0.29 | (3.83, 4.96) |
| 34 | 590.43 | 26.69 | (538.13, 642.74) | 4.56 | 0.29 | (4.00, 5.12) |
| 35 | 619.43 | 26.54 | (567.41, 671.45) | 4.74 | 0.28 | (4.18, 5.30) |
| 36 | 649.62 | 26.39 | (597.90, 701.34) | 4.94 | 0.28 | (4.38, 5.49) |
| 37 | 680.85 | 26.23 | (629.44, 732.27) | 5.15 | 0.28 | (4.60, 5.70) |
| 38 | 712.97 | 26.08 | (661.86, 764.08) | 5.38 | 0.28 | (4.83, 5.93) |
| 39 | 745.84 | 25.93 | (695.02, 796.66) | 5.62 | 0.28 | (5.07, 6.16) |
| 40 | 779.37 | 25.80 | (728.81, 829.94) | 5.85 | 0.28 | (5.31, 6.39) |

| **Table S5.** Weekly effect estimates under the 1-week lag models; NICHD Fetal Growth Studies - Singletons and Fetal 3D Study. | | | | | | |
| --- | --- | --- | --- | --- | --- | --- |
|  | Estimated Fetal Weight (EFW) | | | Fractional Fetal Arm Volume (AVol) | | |
| Gestational Age, week | Estimate | Standard Error | 95% Confidence Interval | Estimate | Standard Error | 95% Confidence Interval |
| 15 | 418.30 | 27.23 | (364.94, 471.66) | 3.97 | 0.29 | (3.39, 4.55) |
| 16 | 419.27 | 27.30 | (365.77, 472.77) | 3.97 | 0.30 | (3.39, 4.55) |
| 17 | 420.60 | 27.40 | (366.89, 474.30) | 3.96 | 0.30 | (3.38, 4.54) |
| 18 | 422.26 | 27.52 | (368.31, 476.20) | 3.96 | 0.30 | (3.38, 4.54) |
| 19 | 424.24 | 27.64 | (370.06, 478.41) | 3.95 | 0.30 | (3.37, 4.54) |
| 20 | 426.61 | 27.75 | (372.23, 480.99) | 3.95 | 0.30 | (3.36, 4.54) |
| 21 | 429.53 | 27.82 | (374.99, 484.06) | 3.95 | 0.30 | (3.36, 4.54) |
| 22 | 433.18 | 27.87 | (378.55, 487.81) | 3.95 | 0.30 | (3.36, 4.54) |
| 23 | 437.83 | 27.89 | (383.17, 492.48) | 3.95 | 0.30 | (3.36, 4.54) |
| 24 | 443.73 | 27.87 | (389.10, 498.35) | 3.96 | 0.30 | (3.38, 4.55) |
| 25 | 451.16 | 27.82 | (396.63, 505.69) | 3.98 | 0.30 | (3.39, 4.57) |
| 26 | 460.41 | 27.75 | (406.03, 514.79) | 4.01 | 0.30 | (3.42, 4.59) |
| 27 | 471.74 | 27.65 | (417.54, 525.94) | 4.05 | 0.30 | (3.47, 4.63) |
| 28 | 485.41 | 27.54 | (431.43, 539.39) | 4.10 | 0.30 | (3.52, 4.68) |
| 29 | 501.63 | 27.42 | (447.89, 555.37) | 4.17 | 0.29 | (3.59, 4.75) |
| 30 | 520.47 | 27.29 | (466.97, 573.96) | 4.26 | 0.29 | (3.69, 4.84) |
| 31 | 541.82 | 27.17 | (488.57, 595.06) | 4.37 | 0.29 | (3.80, 4.94) |
| 32 | 565.52 | 27.04 | (512.53, 618.51) | 4.49 | 0.29 | (3.92, 5.06) |
| 33 | 591.30 | 26.90 | (538.57, 644.03) | 4.64 | 0.29 | (4.07, 5.20) |
| 34 | 618.81 | 26.77 | (566.35, 671.28) | 4.80 | 0.29 | (4.24, 5.36) |
| 35 | 647.62 | 26.63 | (595.43, 699.81) | 4.98 | 0.29 | (4.42, 5.54) |
| 36 | 677.61 | 26.48 | (625.70, 729.52) | 5.18 | 0.28 | (4.62, 5.73) |
| 37 | 708.79 | 26.33 | (657.18, 760.40) | 5.39 | 0.28 | (4.84, 5.94) |
| 38 | 741.08 | 26.17 | (689.78, 792.39) | 5.62 | 0.28 | (5.07, 6.17) |
| 39 | 774.41 | 26.02 | (723.41, 825.41) | 5.86 | 0.28 | (5.31, 6.40) |
| 40 | 808.75 | 25.87 | (758.04, 859.46) | 6.10 | 0.28 | (5.56, 6.64) |

| **Table S6.** Weekly effect estimates under the 2-week lag models; NICHD Fetal Growth Studies - Singletons and Fetal 3D Study. | | | | | | |
| --- | --- | --- | --- | --- | --- | --- |
|  | Estimated Fetal Weight (EFW) | | | Fractional Fetal Arm Volume (AVol) | | |
| Gestational Age, week | Estimate | Standard Error | 95% Confidence Interval | Estimate | Standard Error | 95% Confidence Interval |
| 15 | 443.79 | 27.19 | (390.50, 497.09) | 4.19 | 0.29 | (3.61, 4.77) |
| 16 | 444.73 | 27.22 | (391.37, 498.08) | 4.19 | 0.29 | (3.61, 4.76) |
| 17 | 446.15 | 27.29 | (392.66, 499.64) | 4.18 | 0.30 | (3.60, 4.76) |
| 18 | 448.06 | 27.39 | (394.37, 501.75) | 4.18 | 0.30 | (3.60, 4.76) |
| 19 | 450.42 | 27.51 | (396.50, 504.35) | 4.18 | 0.30 | (3.60, 4.76) |
| 20 | 453.22 | 27.63 | (399.07, 507.37) | 4.18 | 0.30 | (3.60, 4.76) |
| 21 | 456.54 | 27.73 | (402.19, 510.89) | 4.18 | 0.30 | (3.59, 4.77) |
| 22 | 460.53 | 27.81 | (406.03, 515.03) | 4.18 | 0.30 | (3.60, 4.77) |
| 23 | 465.40 | 27.85 | (410.81, 519.99) | 4.19 | 0.30 | (3.60, 4.78) |
| 24 | 471.41 | 27.86 | (416.80, 526.02) | 4.20 | 0.30 | (3.61, 4.79) |
| 25 | 478.84 | 27.84 | (424.27, 533.41) | 4.22 | 0.30 | (3.63, 4.81) |
| 26 | 487.97 | 27.79 | (433.50, 542.45) | 4.25 | 0.30 | (3.66, 4.83) |
| 27 | 499.11 | 27.72 | (444.79, 553.43) | 4.28 | 0.30 | (3.70, 4.87) |
| 28 | 512.54 | 27.62 | (458.41, 566.67) | 4.33 | 0.30 | (3.75, 4.92) |
| 29 | 528.53 | 27.51 | (474.62, 582.45) | 4.40 | 0.30 | (3.82, 4.98) |
| 30 | 547.20 | 27.39 | (493.52, 600.87) | 4.49 | 0.29 | (3.91, 5.06) |
| 31 | 568.46 | 27.26 | (515.03, 621.89) | 4.59 | 0.29 | (4.02, 5.17) |
| 32 | 592.11 | 27.13 | (538.93, 645.29) | 4.72 | 0.29 | (4.15, 5.29) |
| 33 | 617.86 | 27.00 | (564.94, 670.78) | 4.86 | 0.29 | (4.29, 5.43) |
| 34 | 645.31 | 26.87 | (592.65, 697.97) | 5.02 | 0.29 | (4.46, 5.59) |
| 35 | 674.00 | 26.73 | (621.60, 726.39) | 5.20 | 0.29 | (4.64, 5.76) |
| 36 | 703.78 | 26.59 | (651.66, 755.90) | 5.39 | 0.29 | (4.84, 5.95) |
| 37 | 734.75 | 26.45 | (682.91, 786.58) | 5.61 | 0.28 | (5.05, 6.16) |
| 38 | 767.02 | 26.29 | (715.48, 818.55) | 5.83 | 0.28 | (5.28, 6.39) |
| 39 | 800.59 | 26.14 | (749.36, 851.81) | 6.07 | 0.28 | (5.52, 6.62) |
| 40 | 835.48 | 25.98 | (784.55, 886.40) | 6.32 | 0.28 | (5.78, 6.87) |

| **Table S7.** Weekly effect estimates under the 3-week lag models; NICHD Fetal Growth Studies - Singletons and Fetal 3D Study. | | | | | | |
| --- | --- | --- | --- | --- | --- | --- |
|  | Estimated Fetal Weight (EFW) | | | Fractional Fetal Arm Volume (AVol) | | |
| Gestational Age, week | Estimate | Standard Error | 95% Confidence Interval | Estimate | Standard Error | 95% Confidence Interval |
| 15 | 468.23 | 27.23 | (414.86, 521.61) | 4.40 | 0.29 | (3.82, 4.98) |
| 16 | 468.97 | 27.22 | (415.63, 522.31) | 4.39 | 0.29 | (3.81, 4.97) |
| 17 | 470.31 | 27.25 | (416.91, 523.71) | 4.39 | 0.29 | (3.81, 4.96) |
| 18 | 472.27 | 27.31 | (418.73, 525.80) | 4.39 | 0.30 | (3.81, 4.96) |
| 19 | 474.83 | 27.41 | (421.10, 528.56) | 4.39 | 0.30 | (3.81, 4.97) |
| 20 | 477.96 | 27.53 | (424.00, 531.92) | 4.39 | 0.30 | (3.81, 4.97) |
| 21 | 481.67 | 27.64 | (427.49, 535.86) | 4.39 | 0.30 | (3.81, 4.98) |
| 22 | 486.05 | 27.74 | (431.67, 540.43) | 4.40 | 0.30 | (3.81, 4.99) |
| 23 | 491.24 | 27.82 | (436.72, 545.77) | 4.41 | 0.30 | (3.82, 5.00) |
| 24 | 497.48 | 27.86 | (442.88, 552.08) | 4.42 | 0.30 | (3.83, 5.01) |
| 25 | 505.02 | 27.87 | (450.40, 559.64) | 4.44 | 0.30 | (3.85, 5.03) |
| 26 | 514.16 | 27.84 | (459.59, 568.74) | 4.47 | 0.30 | (3.88, 5.05) |
| 27 | 525.21 | 27.79 | (470.74, 579.68) | 4.50 | 0.30 | (3.92, 5.09) |
| 28 | 538.48 | 27.71 | (484.16, 592.80) | 4.55 | 0.30 | (3.97, 5.14) |
| 29 | 554.28 | 27.61 | (500.15, 608.40) | 4.62 | 0.30 | (4.04, 5.20) |
| 30 | 572.77 | 27.50 | (518.87, 626.67) | 4.70 | 0.30 | (4.12, 5.28) |
| 31 | 593.92 | 27.38 | (540.26, 647.58) | 4.81 | 0.29 | (4.23, 5.38) |
| 32 | 617.53 | 27.25 | (564.12, 670.94) | 4.93 | 0.29 | (4.36, 5.50) |
| 33 | 643.27 | 27.12 | (590.11, 696.43) | 5.07 | 0.29 | (4.50, 5.64) |
| 34 | 670.71 | 26.99 | (617.81, 723.61) | 5.23 | 0.29 | (4.67, 5.80) |
| 35 | 699.33 | 26.86 | (646.69, 751.98) | 5.41 | 0.29 | (4.84, 5.97) |
| 36 | 728.97 | 26.72 | (676.60, 781.35) | 5.60 | 0.29 | (5.04, 6.16) |
| 37 | 759.71 | 26.58 | (707.60, 811.81) | 5.81 | 0.29 | (5.25, 6.37) |
| 38 | 791.74 | 26.44 | (739.93, 843.56) | 6.04 | 0.28 | (5.48, 6.59) |
| 39 | 825.31 | 26.28 | (773.80, 876.83) | 6.27 | 0.28 | (5.72, 6.83) |
| 40 | 860.51 | 26.12 | (809.31, 911.71) | 6.53 | 0.28 | (5.98, 7.07) |

| **Table S8.** Weekly effect estimates under the 4-week lag models; NICHD Fetal Growth Studies - Singletons and Fetal 3D Study. | | | | | | |
| --- | --- | --- | --- | --- | --- | --- |
|  | Estimated Fetal Weight (EFW) | | | Fractional Fetal Arm Volume (AVol) | | |
| Gestational Age, week | Estimate | Standard Error | 95% Confidence Interval | Estimate | Standard Error | 95% Confidence Interval |
| 15 | 492.17 | 27.35 | (438.56, 545.77) | 4.60 | 0.30 | (4.02, 5.18) |
| 16 | 492.58 | 27.29 | (439.10, 546.06) | 4.59 | 0.29 | (4.01, 5.17) |
| 17 | 493.68 | 27.27 | (440.23, 547.13) | 4.58 | 0.29 | (4.01, 5.16) |
| 18 | 495.50 | 27.30 | (441.99, 549.01) | 4.58 | 0.29 | (4.00, 5.16) |
| 19 | 498.05 | 27.37 | (444.41, 551.69) | 4.58 | 0.30 | (4.00, 5.16) |
| 20 | 501.34 | 27.46 | (447.51, 555.17) | 4.59 | 0.30 | (4.00, 5.17) |
| 21 | 505.35 | 27.58 | (451.29, 559.40) | 4.59 | 0.30 | (4.01, 5.18) |
| 22 | 510.09 | 27.69 | (455.81, 564.36) | 4.60 | 0.30 | (4.02, 5.19) |
| 23 | 515.65 | 27.79 | (461.18, 570.11) | 4.61 | 0.30 | (4.03, 5.20) |
| 24 | 522.19 | 27.86 | (467.59, 576.80) | 4.63 | 0.30 | (4.04, 5.22) |
| 25 | 529.96 | 27.90 | (475.28, 584.64) | 4.65 | 0.30 | (4.06, 5.24) |
| 26 | 539.22 | 27.90 | (484.53, 593.91) | 4.68 | 0.30 | (4.09, 5.27) |
| 27 | 550.29 | 27.88 | (495.65, 604.93) | 4.71 | 0.30 | (4.13, 5.30) |
| 28 | 563.49 | 27.82 | (508.96, 618.02) | 4.76 | 0.30 | (4.18, 5.35) |
| 29 | 579.17 | 27.74 | (524.79, 633.54) | 4.83 | 0.30 | (4.24, 5.41) |
| 30 | 597.52 | 27.64 | (543.34, 651.70) | 4.91 | 0.30 | (4.33, 5.49) |
| 31 | 618.56 | 27.53 | (564.61, 672.52) | 5.01 | 0.30 | (4.43, 5.59) |
| 32 | 642.13 | 27.40 | (588.42, 695.84) | 5.13 | 0.29 | (4.56, 5.71) |
| 33 | 667.88 | 27.28 | (614.43, 721.34) | 5.28 | 0.29 | (4.70, 5.85) |
| 34 | 695.34 | 27.14 | (642.14, 748.55) | 5.43 | 0.29 | (4.86, 6.01) |
| 35 | 723.97 | 27.01 | (671.02, 776.91) | 5.61 | 0.29 | (5.04, 6.18) |
| 36 | 753.55 | 26.88 | (700.86, 806.23) | 5.80 | 0.29 | (5.24, 6.37) |
| 37 | 784.12 | 26.74 | (731.70, 836.54) | 6.01 | 0.29 | (5.45, 6.57) |
| 38 | 815.89 | 26.60 | (763.75, 868.03) | 6.23 | 0.29 | (5.67, 6.79) |
| 39 | 849.21 | 26.46 | (797.35, 901.06) | 6.47 | 0.28 | (5.91, 7.02) |
| 40 | 884.43 | 26.30 | (832.88, 935.98) | 6.72 | 0.28 | (6.17, 7.27) |

| **Table S9.** Weekly effect estimates under the 5-week lag models; NICHD Fetal Growth Studies - Singletons and Fetal 3D Study. | | | | | | |
| --- | --- | --- | --- | --- | --- | --- |
|  | Estimated Fetal Weight (EFW) | | | Fractional Fetal Arm Volume (AVol) | | |
| Gestational Age, week | Estimate | Standard Error | 95% Confidence Interval | Estimate | Standard Error | 95% Confidence Interval |
| 15 | 515.56 | 27.55 | (461.55, 569.56) | 4.79 | 0.30 | (4.21, 5.38) |
| 16 | 515.55 | 27.44 | (461.77, 569.33) | 4.78 | 0.30 | (4.20, 5.36) |
| 17 | 516.29 | 27.37 | (462.64, 569.94) | 4.77 | 0.30 | (4.19, 5.35) |
| 18 | 517.82 | 27.36 | (464.20, 571.44) | 4.77 | 0.30 | (4.19, 5.35) |
| 19 | 520.19 | 27.39 | (466.52, 573.87) | 4.77 | 0.30 | (4.19, 5.35) |
| 20 | 523.41 | 27.45 | (469.61, 577.22) | 4.77 | 0.30 | (4.19, 5.35) |
| 21 | 527.52 | 27.55 | (473.52, 581.51) | 4.78 | 0.30 | (4.19, 5.36) |
| 22 | 532.51 | 27.66 | (478.30, 586.72) | 4.79 | 0.30 | (4.20, 5.37) |
| 23 | 538.40 | 27.77 | (483.97, 592.83) | 4.80 | 0.30 | (4.22, 5.39) |
| 24 | 545.29 | 27.86 | (490.67, 599.90) | 4.82 | 0.30 | (4.23, 5.41) |
| 25 | 553.35 | 27.93 | (498.60, 608.10) | 4.85 | 0.30 | (4.26, 5.44) |
| 26 | 562.83 | 27.97 | (508.01, 617.65) | 4.87 | 0.30 | (4.28, 5.46) |
| 27 | 574.03 | 27.97 | (519.20, 628.85) | 4.91 | 0.30 | (4.32, 5.50) |
| 28 | 587.27 | 27.94 | (532.50, 642.04) | 4.96 | 0.30 | (4.37, 5.55) |
| 29 | 602.91 | 27.89 | (548.25, 657.57) | 5.02 | 0.30 | (4.43, 5.61) |
| 30 | 621.19 | 27.80 | (566.69, 675.68) | 5.10 | 0.30 | (4.52, 5.69) |
| 31 | 642.16 | 27.70 | (587.86, 696.45) | 5.20 | 0.30 | (4.62, 5.79) |
| 32 | 665.70 | 27.59 | (611.63, 719.76) | 5.33 | 0.30 | (4.75, 5.91) |
| 33 | 691.49 | 27.46 | (637.66, 745.31) | 5.47 | 0.29 | (4.89, 6.05) |
| 34 | 719.01 | 27.33 | (665.44, 772.58) | 5.63 | 0.29 | (5.05, 6.20) |
| 35 | 747.70 | 27.20 | (694.38, 801.01) | 5.80 | 0.29 | (5.23, 6.37) |
| 36 | 777.29 | 27.07 | (724.24, 830.34) | 5.99 | 0.29 | (5.43, 6.56) |
| 37 | 807.80 | 26.93 | (755.01, 860.59) | 6.20 | 0.29 | (5.63, 6.77) |
| 38 | 839.39 | 26.80 | (786.87, 891.92) | 6.42 | 0.29 | (5.86, 6.98) |
| 39 | 872.41 | 26.66 | (820.16, 924.66) | 6.65 | 0.29 | (6.09, 7.21) |
| 40 | 907.36 | 26.51 | (855.40, 959.31) | 6.90 | 0.28 | (6.34, 7.46) |

| **Table S10.** Weekly effect estimates under the 6-week lag models; NICHD Fetal Growth Studies - Singletons and Fetal 3D Study. | | | | | | |
| --- | --- | --- | --- | --- | --- | --- |
|  | Estimated Fetal Weight (EFW) | | | Fractional Fetal Arm Volume (AVol) | | |
| Gestational Age, week | Estimate | Standard Error | 95% Confidence Interval | Estimate | Standard Error | 95% Confidence Interval |
| 15 | 537.55 | 27.84 | (482.98, 592.12) | 4.98 | 0.30 | (4.39, 5.56) |
| 16 | 537.08 | 27.67 | (482.85, 591.32) | 4.96 | 0.30 | (4.37, 5.54) |
| 17 | 537.38 | 27.56 | (483.37, 591.39) | 4.95 | 0.30 | (4.36, 5.53) |
| 18 | 538.51 | 27.49 | (484.63, 592.40) | 4.94 | 0.30 | (4.35, 5.52) |
| 19 | 540.55 | 27.48 | (486.70, 594.40) | 4.93 | 0.30 | (4.35, 5.51) |
| 20 | 543.53 | 27.50 | (489.62, 597.44) | 4.93 | 0.30 | (4.35, 5.52) |
| 21 | 547.51 | 27.57 | (493.47, 601.54) | 4.94 | 0.30 | (4.36, 5.52) |
| 22 | 552.53 | 27.66 | (498.31, 606.75) | 4.95 | 0.30 | (4.37, 5.54) |
| 23 | 558.62 | 27.78 | (504.18, 613.06) | 4.97 | 0.30 | (4.38, 5.56) |
| 24 | 565.80 | 27.88 | (511.15, 620.45) | 4.99 | 0.30 | (4.40, 5.58) |
| 25 | 574.17 | 27.98 | (519.34, 629.01) | 5.02 | 0.30 | (4.43, 5.61) |
| 26 | 583.93 | 28.04 | (528.97, 638.90) | 5.05 | 0.30 | (4.46, 5.64) |
| 27 | 595.35 | 28.08 | (540.32, 650.37) | 5.09 | 0.30 | (4.50, 5.68) |
| 28 | 608.72 | 28.08 | (553.70, 663.75) | 5.14 | 0.30 | (4.55, 5.73) |
| 29 | 624.43 | 28.05 | (569.46, 679.40) | 5.20 | 0.30 | (4.61, 5.79) |
| 30 | 642.71 | 27.98 | (587.86, 697.56) | 5.28 | 0.30 | (4.69, 5.87) |
| 31 | 663.66 | 27.90 | (608.97, 718.34) | 5.38 | 0.30 | (4.79, 5.97) |
| 32 | 687.20 | 27.80 | (632.72, 741.68) | 5.50 | 0.30 | (4.92, 6.09) |
| 33 | 713.06 | 27.68 | (658.81, 767.31) | 5.64 | 0.30 | (5.06, 6.22) |
| 34 | 740.71 | 27.55 | (686.71, 794.71) | 5.80 | 0.30 | (5.22, 6.38) |
| 35 | 769.53 | 27.42 | (715.79, 823.27) | 5.98 | 0.29 | (5.40, 6.55) |
| 36 | 799.23 | 27.29 | (745.74, 852.71) | 6.17 | 0.29 | (5.60, 6.74) |
| 37 | 829.77 | 27.16 | (776.55, 883.00) | 6.38 | 0.29 | (5.81, 6.95) |
| 38 | 861.30 | 27.02 | (808.34, 914.26) | 6.59 | 0.29 | (6.03, 7.16) |
| 39 | 894.13 | 26.88 | (841.44, 946.82) | 6.83 | 0.29 | (6.26, 7.39) |
| 40 | 928.75 | 26.74 | (876.34, 981.16) | 7.07 | 0.29 | (6.51, 7.63) |

| **Table S11.** Weekly effect estimates under the 7-week lag models; NICHD Fetal Growth Studies - Singletons and Fetal 3D Study. | | | | | | |
| --- | --- | --- | --- | --- | --- | --- |
|  | Estimated Fetal Weight (EFW) | | | Fractional Fetal Arm Volume (AVol) | | |
| Gestational Age, week | Estimate | Standard Error | 95% Confidence Interval | Estimate | Standard Error | 95% Confidence Interval |
| 15 | 556.16 | 28.22 | (500.86, 611.47) | 5.13 | 0.30 | (4.53, 5.72) |
| 16 | 555.27 | 27.99 | (500.41, 610.13) | 5.11 | 0.30 | (4.51, 5.70) |
| 17 | 555.10 | 27.82 | (500.57, 609.63) | 5.09 | 0.30 | (4.50, 5.68) |
| 18 | 555.77 | 27.71 | (501.47, 610.08) | 5.08 | 0.30 | (4.49, 5.66) |
| 19 | 557.38 | 27.64 | (503.20, 611.55) | 5.07 | 0.30 | (4.48, 5.65) |
| 20 | 559.98 | 27.62 | (505.84, 614.13) | 5.07 | 0.30 | (4.48, 5.65) |
| 21 | 563.66 | 27.65 | (509.46, 617.85) | 5.07 | 0.30 | (4.49, 5.66) |
| 22 | 568.48 | 27.72 | (514.15, 622.80) | 5.08 | 0.30 | (4.50, 5.67) |
| 23 | 574.52 | 27.81 | (520.02, 629.03) | 5.10 | 0.30 | (4.51, 5.69) |
| 24 | 581.83 | 27.92 | (527.11, 636.56) | 5.12 | 0.30 | (4.53, 5.71) |
| 25 | 590.44 | 28.03 | (535.51, 645.37) | 5.15 | 0.30 | (4.56, 5.74) |
| 26 | 600.47 | 28.12 | (545.36, 655.58) | 5.18 | 0.30 | (4.59, 5.78) |
| 27 | 612.13 | 28.18 | (556.90, 667.37) | 5.23 | 0.30 | (4.63, 5.82) |
| 28 | 625.72 | 28.21 | (570.42, 681.01) | 5.28 | 0.30 | (4.68, 5.87) |
| 29 | 641.57 | 28.21 | (586.27, 696.86) | 5.34 | 0.30 | (4.75, 5.93) |
| 30 | 659.94 | 28.18 | (604.71, 715.16) | 5.42 | 0.30 | (4.83, 6.01) |
| 31 | 680.94 | 28.11 | (625.84, 736.05) | 5.52 | 0.30 | (4.93, 6.11) |
| 32 | 704.55 | 28.03 | (649.62, 759.48) | 5.64 | 0.30 | (5.05, 6.23) |
| 33 | 730.51 | 27.92 | (675.78, 785.23) | 5.78 | 0.30 | (5.19, 6.37) |
| 34 | 758.35 | 27.80 | (703.86, 812.83) | 5.94 | 0.30 | (5.36, 6.53) |
| 35 | 787.40 | 27.67 | (733.16, 841.64) | 6.12 | 0.30 | (5.54, 6.70) |
| 36 | 817.31 | 27.54 | (763.33, 871.29) | 6.31 | 0.30 | (5.73, 6.89) |
| 37 | 848.01 | 27.41 | (794.29, 901.73) | 6.52 | 0.29 | (5.94, 7.09) |
| 38 | 879.61 | 27.27 | (826.15, 933.07) | 6.74 | 0.29 | (6.17, 7.31) |
| 39 | 912.39 | 27.14 | (859.20, 965.58) | 6.97 | 0.29 | (6.40, 7.54) |
| 40 | 946.81 | 27.00 | (893.89, 999.73) | 7.21 | 0.29 | (6.64, 7.78) |

| **Table S12.** Weekly effect estimates under the 8-week lag models; NICHD Fetal Growth Studies - Singletons and Fetal 3D Study. | | | | | | |
| --- | --- | --- | --- | --- | --- | --- |
|  | Estimated Fetal Weight (EFW) | | | Fractional Fetal Arm Volume (AVol) | | |
| Gestational Age, week | Estimate | Standard Error | 95% Confidence Interval | Estimate | Standard Error | 95% Confidence Interval |
| 15 | 568.26 | 28.67 | (512.07, 624.45) | 5.22 | 0.31 | (4.62, 5.83) |
| 16 | 567.08 | 28.39 | (511.44, 622.72) | 5.20 | 0.31 | (4.60, 5.80) |
| 17 | 566.52 | 28.16 | (511.32, 621.71) | 5.18 | 0.30 | (4.58, 5.77) |
| 18 | 566.75 | 27.99 | (511.88, 621.61) | 5.16 | 0.30 | (4.57, 5.75) |
| 19 | 567.89 | 27.88 | (513.25, 622.52) | 5.15 | 0.30 | (4.56, 5.74) |
| 20 | 570.04 | 27.81 | (515.53, 624.55) | 5.15 | 0.30 | (4.56, 5.73) |
| 21 | 573.29 | 27.79 | (518.81, 627.77) | 5.15 | 0.30 | (4.56, 5.73) |
| 22 | 577.75 | 27.82 | (523.22, 632.27) | 5.15 | 0.30 | (4.57, 5.74) |
| 23 | 583.50 | 27.88 | (528.85, 638.15) | 5.17 | 0.30 | (4.58, 5.76) |
| 24 | 590.67 | 27.98 | (535.84, 645.51) | 5.19 | 0.30 | (4.60, 5.78) |
| 25 | 599.32 | 28.09 | (544.27, 654.37) | 5.22 | 0.30 | (4.63, 5.81) |
| 26 | 609.51 | 28.19 | (554.25, 664.76) | 5.25 | 0.30 | (4.66, 5.85) |
| 27 | 621.38 | 28.28 | (565.95, 676.81) | 5.30 | 0.30 | (4.70, 5.89) |
| 28 | 635.18 | 28.34 | (579.63, 690.73) | 5.35 | 0.30 | (4.75, 5.95) |
| 29 | 651.22 | 28.37 | (595.61, 706.83) | 5.42 | 0.30 | (4.82, 6.01) |
| 30 | 669.74 | 28.37 | (614.14, 725.34) | 5.50 | 0.30 | (4.90, 6.09) |
| 31 | 690.88 | 28.33 | (635.35, 746.41) | 5.60 | 0.30 | (5.00, 6.19) |
| 32 | 714.62 | 28.27 | (659.22, 770.02) | 5.72 | 0.30 | (5.12, 6.31) |
| 33 | 740.74 | 28.18 | (685.51, 795.96) | 5.86 | 0.30 | (5.27, 6.45) |
| 34 | 768.80 | 28.07 | (713.79, 823.81) | 6.02 | 0.30 | (5.43, 6.61) |
| 35 | 798.18 | 27.95 | (743.41, 852.96) | 6.20 | 0.30 | (5.61, 6.79) |
| 36 | 828.45 | 27.82 | (773.94, 882.97) | 6.40 | 0.30 | (5.81, 6.98) |
| 37 | 859.47 | 27.68 | (805.21, 913.73) | 6.60 | 0.30 | (6.02, 7.18) |
| 38 | 891.30 | 27.55 | (837.30, 945.29) | 6.82 | 0.29 | (6.25, 7.40) |
| 39 | 924.19 | 27.41 | (870.46, 977.92) | 7.06 | 0.29 | (6.48, 7.63) |
| 40 | 958.58 | 27.28 | (905.12, 1012.05) | 7.30 | 0.29 | (6.72, 7.87) |

| **Table S13.** Weekly effect estimates under the 9-week lag models; NICHD Fetal Growth Studies - Singletons and Fetal 3D Study. | | | | | | |
| --- | --- | --- | --- | --- | --- | --- |
|  | Estimated Fetal Weight (EFW) | | | Fractional Fetal Arm Volume (AVol) | | |
| Gestational Age, week | Estimate | Standard Error | 95% Confidence Interval | Estimate | Standard Error | 95% Confidence Interval |
| 15 | 569.52 | 29.18 | (512.33, 626.71) | 5.22 | 0.31 | (4.61, 5.84) |
| 16 | 568.35 | 28.84 | (511.82, 624.88) | 5.20 | 0.31 | (4.59, 5.81) |
| 17 | 567.61 | 28.56 | (511.63, 623.58) | 5.18 | 0.31 | (4.57, 5.78) |
| 18 | 567.53 | 28.33 | (511.99, 623.06) | 5.16 | 0.31 | (4.56, 5.76) |
| 19 | 568.27 | 28.16 | (513.07, 623.47) | 5.15 | 0.30 | (4.55, 5.74) |
| 20 | 569.98 | 28.05 | (515.01, 624.95) | 5.14 | 0.30 | (4.54, 5.73) |
| 21 | 572.77 | 27.98 | (517.93, 627.62) | 5.13 | 0.30 | (4.54, 5.72) |
| 22 | 576.77 | 27.96 | (521.96, 631.58) | 5.14 | 0.30 | (4.55, 5.73) |
| 23 | 582.09 | 27.99 | (527.23, 636.95) | 5.15 | 0.30 | (4.56, 5.74) |
| 24 | 588.88 | 28.05 | (533.89, 643.86) | 5.17 | 0.30 | (4.57, 5.76) |
| 25 | 597.27 | 28.15 | (542.11, 652.44) | 5.19 | 0.30 | (4.60, 5.79) |
| 26 | 607.39 | 28.25 | (552.01, 662.77) | 5.23 | 0.30 | (4.63, 5.82) |
| 27 | 619.32 | 28.36 | (563.73, 674.90) | 5.27 | 0.31 | (4.67, 5.87) |
| 28 | 633.24 | 28.45 | (577.49, 689.00) | 5.33 | 0.31 | (4.73, 5.93) |
| 29 | 649.44 | 28.50 | (593.57, 705.31) | 5.39 | 0.31 | (4.79, 5.99) |
| 30 | 668.13 | 28.53 | (612.20, 724.05) | 5.48 | 0.31 | (4.88, 6.08) |
| 31 | 689.44 | 28.53 | (633.52, 745.35) | 5.58 | 0.31 | (4.98, 6.18) |
| 32 | 713.36 | 28.49 | (657.52, 769.19) | 5.70 | 0.31 | (5.10, 6.30) |
| 33 | 739.70 | 28.42 | (684.00, 795.40) | 5.84 | 0.30 | (5.24, 6.44) |
| 34 | 768.05 | 28.33 | (712.53, 823.58) | 6.00 | 0.30 | (5.41, 6.60) |
| 35 | 797.82 | 28.22 | (742.51, 853.12) | 6.19 | 0.30 | (5.59, 6.78) |
| 36 | 828.59 | 28.09 | (773.52, 883.65) | 6.38 | 0.30 | (5.79, 6.97) |
| 37 | 860.12 | 27.96 | (805.31, 914.92) | 6.60 | 0.30 | (6.01, 7.18) |
| 38 | 892.38 | 27.83 | (837.83, 946.92) | 6.82 | 0.30 | (6.24, 7.40) |
| 39 | 925.59 | 27.69 | (871.31, 979.87) | 7.05 | 0.30 | (6.47, 7.63) |
| 40 | 960.17 | 27.56 | (906.16, 1014.18) | 7.30 | 0.29 | (6.72, 7.87) |

| **Table S14.** Weekly effect estimates under the 10-week lag models; NICHD Fetal Growth Studies - Singletons and Fetal 3D Study. | | | | | | |
| --- | --- | --- | --- | --- | --- | --- |
|  | Estimated Fetal Weight (EFW) | | | Fractional Fetal Arm Volume (AVol) | | |
| Gestational Age, week | Estimate | Standard Error | 95% Confidence Interval | Estimate | Standard Error | 95% Confidence Interval |
| 15 | 554.57 | 29.71 | (496.34, 612.80) | 5.08 | 0.32 | (4.46, 5.71) |
| 16 | 553.91 | 29.32 | (496.44, 611.38) | 5.07 | 0.32 | (4.45, 5.69) |
| 17 | 553.39 | 28.99 | (496.58, 610.20) | 5.05 | 0.31 | (4.44, 5.66) |
| 18 | 553.30 | 28.70 | (497.04, 609.55) | 5.03 | 0.31 | (4.42, 5.64) |
| 19 | 553.87 | 28.48 | (498.05, 609.68) | 5.01 | 0.31 | (4.41, 5.62) |
| 20 | 555.28 | 28.31 | (499.80, 610.76) | 5.00 | 0.31 | (4.40, 5.60) |
| 21 | 557.68 | 28.19 | (502.43, 612.93) | 4.99 | 0.30 | (4.40, 5.59) |
| 22 | 561.23 | 28.12 | (506.11, 616.36) | 4.99 | 0.30 | (4.40, 5.59) |
| 23 | 566.07 | 28.11 | (510.98, 621.16) | 5.00 | 0.30 | (4.41, 5.60) |
| 24 | 572.36 | 28.13 | (517.22, 627.49) | 5.02 | 0.30 | (4.42, 5.61) |
| 25 | 580.26 | 28.19 | (525.00, 635.52) | 5.04 | 0.30 | (4.44, 5.63) |
| 26 | 589.99 | 28.29 | (534.55, 645.43) | 5.07 | 0.30 | (4.47, 5.67) |
| 27 | 601.71 | 28.39 | (546.05, 657.36) | 5.12 | 0.31 | (4.52, 5.71) |
| 28 | 615.55 | 28.50 | (559.70, 671.41) | 5.17 | 0.31 | (4.57, 5.77) |
| 29 | 631.76 | 28.58 | (575.74, 687.78) | 5.24 | 0.31 | (4.63, 5.84) |
| 30 | 650.53 | 28.64 | (594.40, 706.67) | 5.32 | 0.31 | (4.72, 5.92) |
| 31 | 671.97 | 28.67 | (615.79, 728.16) | 5.42 | 0.31 | (4.82, 6.03) |
| 32 | 696.08 | 28.66 | (639.91, 752.25) | 5.55 | 0.31 | (4.94, 6.15) |
| 33 | 722.68 | 28.62 | (666.59, 778.77) | 5.69 | 0.31 | (5.09, 6.29) |
| 34 | 751.38 | 28.55 | (695.42, 807.33) | 5.85 | 0.31 | (5.25, 6.45) |
| 35 | 781.59 | 28.45 | (725.82, 837.36) | 6.04 | 0.31 | (5.44, 6.64) |
| 36 | 812.93 | 28.34 | (757.38, 868.48) | 6.24 | 0.30 | (5.64, 6.84) |
| 37 | 845.15 | 28.22 | (789.85, 900.46) | 6.46 | 0.30 | (5.87, 7.05) |
| 38 | 878.11 | 28.08 | (823.07, 933.16) | 6.69 | 0.30 | (6.10, 7.28) |
| 39 | 911.90 | 27.95 | (857.12, 966.68) | 6.92 | 0.30 | (6.34, 7.51) |
| 40 | 946.92 | 27.81 | (892.41, 1001.43) | 7.17 | 0.30 | (6.59, 7.75) |

| **Table S15.** Weekly effect estimates under the 11-week lag models; NICHD Fetal Growth Studies - Singletons and Fetal 3D Study. | | | | | | |
| --- | --- | --- | --- | --- | --- | --- |
|  | Estimated Fetal Weight (EFW) | | | Fractional Fetal Arm Volume (AVol) | | |
| Gestational Age, week | Estimate | Standard Error | 95% Confidence Interval | Estimate | Standard Error | 95% Confidence Interval |
| 15 | 518.00 | 30.22 | (458.78, 577.23) | 4.76 | 0.33 | (4.12, 5.40) |
| 16 | 518.58 | 29.78 | (460.20, 576.95) | 4.75 | 0.32 | (4.12, 5.38) |
| 17 | 518.87 | 29.40 | (461.25, 576.49) | 4.74 | 0.32 | (4.12, 5.36) |
| 18 | 519.26 | 29.06 | (462.30, 576.22) | 4.73 | 0.31 | (4.11, 5.34) |
| 19 | 520.06 | 28.78 | (463.65, 576.47) | 4.71 | 0.31 | (4.11, 5.32) |
| 20 | 521.48 | 28.55 | (465.51, 577.45) | 4.70 | 0.31 | (4.10, 5.31) |
| 21 | 523.73 | 28.38 | (468.10, 579.36) | 4.69 | 0.31 | (4.09, 5.30) |
| 22 | 526.99 | 28.27 | (471.58, 582.39) | 4.69 | 0.30 | (4.09, 5.29) |
| 23 | 531.42 | 28.20 | (476.15, 586.70) | 4.70 | 0.30 | (4.10, 5.29) |
| 24 | 537.21 | 28.18 | (481.98, 592.45) | 4.71 | 0.30 | (4.11, 5.30) |
| 25 | 544.56 | 28.21 | (489.27, 599.85) | 4.72 | 0.30 | (4.13, 5.32) |
| 26 | 553.68 | 28.27 | (498.27, 609.09) | 4.75 | 0.30 | (4.16, 5.35) |
| 27 | 564.86 | 28.36 | (509.27, 620.45) | 4.79 | 0.31 | (4.19, 5.39) |
| 28 | 578.33 | 28.47 | (522.53, 634.12) | 4.84 | 0.31 | (4.24, 5.45) |
| 29 | 594.29 | 28.57 | (538.29, 650.29) | 4.91 | 0.31 | (4.31, 5.51) |
| 30 | 612.93 | 28.65 | (556.77, 669.10) | 4.99 | 0.31 | (4.39, 5.60) |
| 31 | 634.36 | 28.71 | (578.09, 690.63) | 5.10 | 0.31 | (4.49, 5.70) |
| 32 | 658.56 | 28.73 | (602.24, 714.88) | 5.22 | 0.31 | (4.61, 5.82) |
| 33 | 685.37 | 28.73 | (629.07, 741.67) | 5.36 | 0.31 | (4.76, 5.97) |
| 34 | 714.41 | 28.68 | (658.19, 770.63) | 5.53 | 0.31 | (4.93, 6.13) |
| 35 | 745.10 | 28.61 | (689.02, 801.18) | 5.72 | 0.31 | (5.12, 6.32) |
| 36 | 777.07 | 28.52 | (721.17, 832.96) | 5.92 | 0.31 | (5.32, 6.52) |
| 37 | 810.07 | 28.40 | (754.40, 865.74) | 6.15 | 0.30 | (5.55, 6.74) |
| 38 | 843.95 | 28.28 | (788.52, 899.37) | 6.38 | 0.30 | (5.79, 6.98) |
| 39 | 878.65 | 28.14 | (823.49, 933.81) | 6.63 | 0.30 | (6.04, 7.22) |
| 40 | 914.42 | 28.01 | (859.53, 969.32) | 6.88 | 0.30 | (6.29, 7.47) |

| **Table S16.** Weekly effect estimates under the 12-week lag models; NICHD Fetal Growth Studies - Singletons and Fetal 3D Study. | | | | | | |
| --- | --- | --- | --- | --- | --- | --- |
|  | Estimated Fetal Weight (EFW) | | | Fractional Fetal Arm Volume (AVol) | | |
| Gestational Age, week | Estimate | Standard Error | 95% Confidence Interval | Estimate | Standard Error | 95% Confidence Interval |
| 15 | 456.21 | 30.65 | (396.13, 516.28) | 4.22 | 0.33 | (3.57, 4.86) |
| 16 | 458.88 | 30.19 | (399.71, 518.05) | 4.23 | 0.33 | (3.59, 4.87) |
| 17 | 460.77 | 29.76 | (402.44, 519.10) | 4.23 | 0.32 | (3.60, 4.86) |
| 18 | 462.33 | 29.37 | (404.76, 519.91) | 4.23 | 0.32 | (3.61, 4.85) |
| 19 | 463.94 | 29.04 | (407.02, 520.86) | 4.22 | 0.31 | (3.61, 4.84) |
| 20 | 465.87 | 28.76 | (409.50, 522.24) | 4.21 | 0.31 | (3.60, 4.82) |
| 21 | 468.37 | 28.53 | (412.44, 524.29) | 4.21 | 0.31 | (3.60, 4.81) |
| 22 | 471.66 | 28.36 | (416.06, 527.25) | 4.21 | 0.31 | (3.60, 4.81) |
| 23 | 475.93 | 28.25 | (420.56, 531.29) | 4.21 | 0.30 | (3.61, 4.80) |
| 24 | 481.39 | 28.18 | (426.15, 536.62) | 4.21 | 0.30 | (3.62, 4.81) |
| 25 | 488.25 | 28.16 | (433.05, 543.45) | 4.23 | 0.30 | (3.63, 4.83) |
| 26 | 496.77 | 28.19 | (441.52, 552.02) | 4.25 | 0.30 | (3.66, 4.85) |
| 27 | 507.23 | 28.25 | (451.86, 562.60) | 4.29 | 0.30 | (3.69, 4.88) |
| 28 | 519.99 | 28.34 | (464.44, 575.54) | 4.33 | 0.31 | (3.74, 4.93) |
| 29 | 535.38 | 28.44 | (479.63, 591.13) | 4.40 | 0.31 | (3.80, 5.00) |
| 30 | 553.58 | 28.55 | (497.63, 609.53) | 4.48 | 0.31 | (3.87, 5.08) |
| 31 | 574.72 | 28.63 | (518.61, 630.83) | 4.58 | 0.31 | (3.97, 5.18) |
| 32 | 598.79 | 28.68 | (542.57, 655.01) | 4.70 | 0.31 | (4.09, 5.30) |
| 33 | 625.64 | 28.71 | (569.37, 681.90) | 4.84 | 0.31 | (4.24, 5.45) |
| 34 | 654.90 | 28.70 | (598.66, 711.15) | 5.01 | 0.31 | (4.41, 5.62) |
| 35 | 686.03 | 28.65 | (629.87, 742.19) | 5.20 | 0.31 | (4.60, 5.80) |
| 36 | 718.62 | 28.58 | (662.60, 774.64) | 5.41 | 0.31 | (4.81, 6.01) |
| 37 | 752.44 | 28.49 | (696.61, 808.27) | 5.64 | 0.31 | (5.04, 6.24) |
| 38 | 787.31 | 28.37 | (731.71, 842.92) | 5.89 | 0.30 | (5.29, 6.48) |
| 39 | 823.18 | 28.25 | (767.82, 878.54) | 6.14 | 0.30 | (5.55, 6.73) |
| 40 | 860.10 | 28.11 | (805.00, 915.19) | 6.40 | 0.30 | (5.81, 6.99) |

| **Table S17.** Weekly effect estimates under the 13-week lag models; NICHD Fetal Growth Studies - Singletons and Fetal 3D Study. | | | | | | |
| --- | --- | --- | --- | --- | --- | --- |
|  | Estimated Fetal Weight (EFW) | | | Fractional Fetal Arm Volume (AVol) | | |
| Gestational Age, week | Estimate | Standard Error | 95% Confidence Interval | Estimate | Standard Error | 95% Confidence Interval |
| 15 | 368.99 | 30.98 | (308.26, 429.71) | 3.44 | 0.33 | (2.79, 4.10) |
| 16 | 374.65 | 30.51 | (314.86, 434.44) | 3.48 | 0.33 | (2.84, 4.13) |
| 17 | 378.98 | 30.05 | (320.08, 437.87) | 3.51 | 0.32 | (2.87, 4.14) |
| 18 | 382.50 | 29.62 | (324.44, 440.56) | 3.52 | 0.32 | (2.89, 4.15) |
| 19 | 385.64 | 29.24 | (328.33, 442.95) | 3.53 | 0.32 | (2.91, 4.15) |
| 20 | 388.72 | 28.91 | (332.06, 445.38) | 3.53 | 0.31 | (2.92, 4.14) |
| 21 | 392.03 | 28.63 | (335.92, 448.14) | 3.53 | 0.31 | (2.93, 4.14) |
| 22 | 395.83 | 28.40 | (340.15, 451.50) | 3.53 | 0.31 | (2.93, 4.14) |
| 23 | 400.34 | 28.24 | (345.00, 455.68) | 3.54 | 0.31 | (2.94, 4.14) |
| 24 | 405.79 | 28.12 | (350.68, 460.91) | 3.55 | 0.30 | (2.95, 4.14) |
| 25 | 412.42 | 28.05 | (357.43, 467.41) | 3.56 | 0.30 | (2.97, 4.15) |
| 26 | 420.49 | 28.04 | (365.54, 475.44) | 3.58 | 0.30 | (2.99, 4.17) |
| 27 | 430.31 | 28.06 | (375.31, 485.31) | 3.61 | 0.30 | (3.01, 4.20) |
| 28 | 442.25 | 28.12 | (387.13, 497.37) | 3.65 | 0.30 | (3.05, 4.24) |
| 29 | 456.76 | 28.21 | (401.47, 512.05) | 3.71 | 0.30 | (3.11, 4.30) |
| 30 | 474.18 | 28.31 | (418.69, 529.68) | 3.78 | 0.31 | (3.18, 4.38) |
| 31 | 494.67 | 28.41 | (438.98, 550.36) | 3.87 | 0.31 | (3.27, 4.48) |
| 32 | 518.26 | 28.50 | (462.41, 574.11) | 3.99 | 0.31 | (3.39, 4.59) |
| 33 | 544.85 | 28.55 | (488.89, 600.81) | 4.14 | 0.31 | (3.53, 4.74) |
| 34 | 574.10 | 28.57 | (518.10, 630.10) | 4.30 | 0.31 | (3.70, 4.91) |
| 35 | 605.47 | 28.56 | (549.49, 661.45) | 4.49 | 0.31 | (3.89, 5.10) |
| 36 | 638.57 | 28.52 | (582.67, 694.47) | 4.71 | 0.31 | (4.11, 5.31) |
| 37 | 673.15 | 28.45 | (617.39, 728.90) | 4.94 | 0.31 | (4.34, 5.54) |
| 38 | 709.02 | 28.35 | (653.45, 764.58) | 5.19 | 0.30 | (4.60, 5.79) |
| 39 | 746.09 | 28.24 | (690.74, 801.44) | 5.46 | 0.30 | (4.86, 6.05) |
| 40 | 784.41 | 28.11 | (729.31, 839.51) | 5.73 | 0.30 | (5.14, 6.32) |

| **Table S18.** Weekly effect estimates under the 14-week lag models; NICHD Fetal Growth Studies - Singletons and Fetal 3D Study. | | | | | | |
| --- | --- | --- | --- | --- | --- | --- |
|  | Estimated Fetal Weight (EFW) | | | Fractional Fetal Arm Volume (AVol) | | |
| Gestational Age, week | Estimate | Standard Error | 95% Confidence Interval | Estimate | Standard Error | 95% Confidence Interval |
| 15 | 259.45 | 31.20 | (198.30, 320.60) | 2.47 | 0.34 | (1.81, 3.13) |
| 16 | 268.78 | 30.73 | (208.54, 329.02) | 2.54 | 0.33 | (1.89, 3.19) |
| 17 | 276.31 | 30.26 | (217.00, 335.63) | 2.59 | 0.33 | (1.95, 3.23) |
| 18 | 282.57 | 29.81 | (224.15, 341.00) | 2.63 | 0.32 | (2.00, 3.26) |
| 19 | 288.00 | 29.38 | (230.40, 345.59) | 2.66 | 0.32 | (2.04, 3.28) |
| 20 | 292.94 | 29.00 | (236.09, 349.79) | 2.68 | 0.31 | (2.06, 3.29) |
| 21 | 297.73 | 28.68 | (241.53, 353.93) | 2.69 | 0.31 | (2.09, 3.30) |
| 22 | 302.64 | 28.40 | (246.98, 358.30) | 2.70 | 0.31 | (2.10, 3.31) |
| 23 | 307.92 | 28.18 | (252.70, 363.15) | 2.71 | 0.30 | (2.12, 3.31) |
| 24 | 313.83 | 28.01 | (258.93, 368.73) | 2.73 | 0.30 | (2.13, 3.32) |
| 25 | 320.62 | 27.89 | (265.94, 375.29) | 2.74 | 0.30 | (2.15, 3.33) |
| 26 | 328.56 | 27.83 | (274.01, 383.10) | 2.76 | 0.30 | (2.17, 3.35) |
| 27 | 337.98 | 27.81 | (283.47, 392.49) | 2.79 | 0.30 | (2.20, 3.38) |
| 28 | 349.25 | 27.83 | (294.70, 403.81) | 2.82 | 0.30 | (2.23, 3.41) |
| 29 | 362.85 | 27.89 | (308.18, 417.52) | 2.87 | 0.30 | (2.28, 3.46) |
| 30 | 379.23 | 27.98 | (324.38, 434.07) | 2.94 | 0.30 | (2.35, 3.53) |
| 31 | 398.71 | 28.09 | (343.66, 453.76) | 3.03 | 0.30 | (2.43, 3.62) |
| 32 | 421.44 | 28.19 | (366.20, 476.69) | 3.14 | 0.30 | (2.54, 3.73) |
| 33 | 447.37 | 28.27 | (391.97, 502.77) | 3.27 | 0.30 | (2.68, 3.87) |
| 34 | 476.22 | 28.32 | (420.71, 531.72) | 3.44 | 0.31 | (2.84, 4.04) |
| 35 | 507.49 | 28.34 | (451.94, 563.05) | 3.63 | 0.31 | (3.03, 4.23) |
| 36 | 540.83 | 28.33 | (485.30, 596.36) | 3.84 | 0.30 | (3.25, 4.44) |
| 37 | 575.97 | 28.29 | (520.52, 631.42) | 4.08 | 0.30 | (3.49, 4.68) |
| 38 | 612.70 | 28.22 | (557.39, 668.01) | 4.34 | 0.30 | (3.75, 4.93) |
| 39 | 650.92 | 28.12 | (595.80, 706.05) | 4.61 | 0.30 | (4.02, 5.21) |
| 40 | 690.65 | 28.01 | (635.74, 745.55) | 4.90 | 0.30 | (4.31, 5.49) |

**Table S19.** Measures of model fit under each of the lag specifications for estimated fetal weight and fractional fetal arm volume; NICHD Fetal 3D Study - Singletons, 2015-2019; NICHD Fetal Growth Studies - Singletons and Fetal 3D Study.

|  | **EFW**^a^ | | **AVol**^b^ | |
| --- | --- | --- | --- | --- |
| Lag Specification | AIC | BIC | AIC | BIC |
| 0-Week | 820269.6 | 820876.6 | 180463.2 | 181054.9 |
| 1-Week | 820222.4 | 820829.4 | 180428.5 | 181020.2 |
| 2-Week | 820182.4 | 820789.4 | 180398.4 | 180990.1 |
| 3-Week | 820146.7 | 820753.7 | 180371.4 | 180963.1 |
| 4-Week | 820113.5 | 820720.5 | 180346.7 | 180938.5 |
| 5-Week | 820082.9 | 820689.9 | 180324.6 | 180916.3 |
| 6-Week | 820057.5 | 820664.6 | 180306.7 | 180898.4 |
| 7-Week | **820043.7**^c^ | **820650.7**^c^ | **180296.9**^c^ | **180888.6**^c^ |
| 8-Week | 820049.2 | 820656.2 | 180300.1 | 180891.8 |
| 9-Week | 820082.3 | 820689.3 | 180321.3 | 180913.0 |
| 10-Week | 820150.2 | 820757.2 | 180364.9 | 180956.6 |
| 11-Week | 820256.9 | 820863.9 | 180433.2 | 181024.9 |
| 12-Week | 820400.5 | 821007.5 | 180524.5 | 181116.2 |
| 13-Week | 820570.9 | 821177.9 | 180631.9 | 181223.6 |
| 14-Week | 820749.3 | 821356.3 | 180742.7 | 181334.4 |

^a^ EFW = estimated fetal weight
^b^ AVol = fractional fetal arm volume
^c^ Optimal values are indicated in bold-face

|  | **Model Fits** | |
| --- | --- | --- |
| Lag Specification | AIC | BIC |
| 0-Week | 180951.8 | 181543.5 |
| 1-Week | 180914.8 | 181506.5 |
| 2-Week | 180882.5 | 181474.2 |
| 3-Week | 180853.4 | 181445.1 |
| 4-Week | 180826.7 | 181418.4 |
| 5-Week | 180802.6 | 181394.3 |
| 6-Week | 180783.0 | 181374.7 |
| 7-Week | **180771.8**^a^ | **181363.5**^a^ |
| 8-Week | 180774.0 | 181365.8 |
| 9-Week | 180795.1 | 181386.8 |
| 10-Week | 180839.4 | 181431.1 |
| 11-Week | 180909.7 | 181501.4 |
| 12-Week | 181004.4 | 181596.1 |
| 13-Week | 181116.3 | 181708.0 |
| 14-Week | 181231.8 | 181823.6 |

**Table S20.** Measures of model fit under each of the lag specifications for fractional fetal arm volume from IPCW sensitivity analysis; NICHD Fetal 3D Study - Singletons, 2015-2019; NICHD Fetal Growth Studies - Singletons and Fetal 3D Study.

**^a^** Optimal values are indicated in bold-face

|  | Fractional Fetal Arm Volume (AVol) | | |
| --- | --- | --- | --- |
| Gestational Age, week | Estimate | Standard Error | 95% Confidence Interval |
| 15 | 3.86 | 0.29 | (3.29, 4.43) |
| 16 | 3.86 | 0.29 | (3.29, 4.43) |
| 17 | 3.85 | 0.29 | (3.28, 4.43) |
| 18 | 3.85 | 0.29 | (3.27, 4.43) |
| 19 | 3.84 | 0.30 | (3.27, 4.42) |
| 20 | 3.84 | 0.30 | (3.26, 4.42) |
| 21 | 3.83 | 0.30 | (3.25, 4.42) |
| 22 | 3.83 | 0.30 | (3.25, 4.41) |
| 23 | 3.83 | 0.30 | (3.25, 4.42) |
| 24 | 3.84 | 0.30 | (3.26, 4.42) |
| 25 | 3.86 | 0.30 | (3.28, 4.44) |
| 26 | 3.88 | 0.29 | (3.30, 4.46) |
| 27 | 3.92 | 0.29 | (3.34, 4.49) |
| 28 | 3.97 | 0.29 | (3.39, 4.54) |
| 29 | 4.03 | 0.29 | (3.46, 4.60) |
| 30 | 4.11 | 0.29 | (3.54, 4.67) |
| 31 | 4.20 | 0.29 | (3.63, 4.76) |
| 32 | 4.31 | 0.29 | (3.75, 4.87) |
| 33 | 4.44 | 0.29 | (3.88, 5.00) |
| 34 | 4.58 | 0.28 | (4.02, 5.14) |
| 35 | 4.74 | 0.28 | (4.19, 5.29) |
| 36 | 4.92 | 0.28 | (4.37, 5.47) |
| 37 | 5.11 | 0.28 | (4.56, 5.65) |
| 38 | 5.31 | 0.28 | (4.76, 5.85) |
| 39 | 5.52 | 0.28 | (4.97, 6.06) |
| 40 | 5.72 | 0.27 | (5.19, 6.26) |

**Table S21.** Weekly effect estimates under the 0-week lag model for fractional fetal arm volume from IPCW sensitivity analysis; NICHD Fetal Growth Studies - Singletons and Fetal 3D Study.

|  | Fractional Fetal Arm Volume (AVol) | | |
| --- | --- | --- | --- |
| Gestational Age, week | Estimate | Standard Error | 95% Confidence Interval |
| 15 | 4.10 | 0.29 | (3.53, 4.67) |
| 16 | 4.10 | 0.29 | (3.53, 4.67) |
| 17 | 4.10 | 0.29 | (3.53, 4.67) |
| 18 | 4.09 | 0.29 | (3.52, 4.67) |
| 19 | 4.09 | 0.29 | (3.52, 4.67) |
| 20 | 4.09 | 0.29 | (3.51, 4.67) |
| 21 | 4.09 | 0.30 | (3.51, 4.67) |
| 22 | 4.09 | 0.30 | (3.51, 4.67) |
| 23 | 4.09 | 0.30 | (3.51, 4.67) |
| 24 | 4.10 | 0.30 | (3.52, 4.68) |
| 25 | 4.12 | 0.30 | (3.54, 4.70) |
| 26 | 4.14 | 0.29 | (3.56, 4.72) |
| 27 | 4.17 | 0.29 | (3.60, 4.75) |
| 28 | 4.22 | 0.29 | (3.64, 4.79) |
| 29 | 4.28 | 0.29 | (3.71, 4.85) |
| 30 | 4.35 | 0.29 | (3.78, 4.92) |
| 31 | 4.45 | 0.29 | (3.88, 5.01) |
| 32 | 4.56 | 0.29 | (3.99, 5.12) |
| 33 | 4.68 | 0.29 | (4.12, 5.24) |
| 34 | 4.82 | 0.28 | (4.26, 5.38) |
| 35 | 4.98 | 0.28 | (4.42, 5.53) |
| 36 | 5.15 | 0.28 | (4.60, 5.70) |
| 37 | 5.34 | 0.28 | (4.79, 5.89) |
| 38 | 5.54 | 0.28 | (5.00, 6.09) |
| 39 | 5.76 | 0.28 | (5.21, 6.30) |
| 40 | 5.97 | 0.28 | (5.43, 6.51) |

**Table S22.** Weekly effect estimates under the 1-week lag model for fractional fetal arm volume from IPCW sensitivity analysis; NICHD Fetal Growth Studies - Singletons and Fetal 3D Study.

|  | Fractional Fetal Arm Volume (AVol) | | |
| --- | --- | --- | --- |
| Gestational Age, week | Estimate | Standard Error | 95% Confidence Interval |
| 15 | 4.32 | 0.29 | (3.75, 4.88) |
| 16 | 4.31 | 0.29 | (3.75, 4.88) |
| 17 | 4.31 | 0.29 | (3.74, 4.88) |
| 18 | 4.31 | 0.29 | (3.74, 4.88) |
| 19 | 4.31 | 0.29 | (3.74, 4.89) |
| 20 | 4.32 | 0.29 | (3.74, 4.89) |
| 21 | 4.32 | 0.29 | (3.74, 4.89) |
| 22 | 4.32 | 0.30 | (3.74, 4.90) |
| 23 | 4.33 | 0.30 | (3.75, 4.91) |
| 24 | 4.34 | 0.30 | (3.76, 4.92) |
| 25 | 4.35 | 0.30 | (3.77, 4.93) |
| 26 | 4.37 | 0.30 | (3.80, 4.95) |
| 27 | 4.41 | 0.29 | (3.83, 4.98) |
| 28 | 4.45 | 0.29 | (3.87, 5.02) |
| 29 | 4.51 | 0.29 | (3.93, 5.08) |
| 30 | 4.58 | 0.29 | (4.01, 5.15) |
| 31 | 4.67 | 0.29 | (4.10, 5.24) |
| 32 | 4.78 | 0.29 | (4.21, 5.35) |
| 33 | 4.90 | 0.29 | (4.34, 5.47) |
| 34 | 5.04 | 0.29 | (4.49, 5.60) |
| 35 | 5.20 | 0.28 | (4.64, 5.76) |
| 36 | 5.37 | 0.28 | (4.82, 5.92) |
| 37 | 5.56 | 0.28 | (5.01, 6.11) |
| 38 | 5.76 | 0.28 | (5.21, 6.31) |
| 39 | 5.97 | 0.28 | (5.43, 6.52) |
| 40 | 6.19 | 0.28 | (5.65, 6.73) |

**Table S23.** Weekly effect estimates under the 2-week lag model for fractional fetal arm volume from IPCW sensitivity analysis; NICHD Fetal Growth Studies - Singletons and Fetal 3D Study.

|  | Fractional Fetal Arm Volume (AVol) | | |
| --- | --- | --- | --- |
| Gestational Age, week | Estimate | Standard Error | 95% Confidence Interval |
| 15 | 4.52 | 0.29 | (3.96, 5.09) |
| 16 | 4.52 | 0.29 | (3.95, 5.08) |
| 17 | 4.51 | 0.29 | (3.95, 5.08) |
| 18 | 4.51 | 0.29 | (3.95, 5.08) |
| 19 | 4.52 | 0.29 | (3.95, 5.09) |
| 20 | 4.52 | 0.29 | (3.95, 5.09) |
| 21 | 4.53 | 0.29 | (3.95, 5.10) |
| 22 | 4.53 | 0.29 | (3.96, 5.11) |
| 23 | 4.54 | 0.30 | (3.96, 5.12) |
| 24 | 4.56 | 0.30 | (3.98, 5.14) |
| 25 | 4.57 | 0.30 | (3.99, 5.15) |
| 26 | 4.59 | 0.30 | (4.01, 5.17) |
| 27 | 4.62 | 0.30 | (4.05, 5.20) |
| 28 | 4.67 | 0.29 | (4.09, 5.24) |
| 29 | 4.72 | 0.29 | (4.15, 5.30) |
| 30 | 4.79 | 0.29 | (4.22, 5.37) |
| 31 | 4.88 | 0.29 | (4.31, 5.45) |
| 32 | 4.99 | 0.29 | (4.42, 5.56) |
| 33 | 5.12 | 0.29 | (4.55, 5.68) |
| 34 | 5.26 | 0.29 | (4.69, 5.82) |
| 35 | 5.41 | 0.29 | (4.85, 5.97) |
| 36 | 5.58 | 0.28 | (5.02, 6.13) |
| 37 | 5.76 | 0.28 | (5.21, 6.31) |
| 38 | 5.96 | 0.28 | (5.41, 6.51) |
| 39 | 6.17 | 0.28 | (5.63, 6.72) |
| 40 | 6.39 | 0.28 | (5.85, 6.94) |

**Table S24.** Weekly effect estimates under the 3-week lag model for fractional fetal arm volume from IPCW sensitivity analysis; NICHD Fetal Growth Studies - Singletons and Fetal 3D Study.

|  | Fractional Fetal Arm Volume (AVol) | | |
| --- | --- | --- | --- |
| Gestational Age, week | Estimate | Standard Error | 95% Confidence Interval |
| 15 | 4.72 | 0.29 | (4.16, 5.29) |
| 16 | 4.71 | 0.29 | (4.15, 5.28) |
| 17 | 4.71 | 0.29 | (4.14, 5.28) |
| 18 | 4.71 | 0.29 | (4.14, 5.27) |
| 19 | 4.71 | 0.29 | (4.14, 5.28) |
| 20 | 4.71 | 0.29 | (4.14, 5.28) |
| 21 | 4.72 | 0.29 | (4.15, 5.30) |
| 22 | 4.73 | 0.29 | (4.16, 5.31) |
| 23 | 4.75 | 0.29 | (4.17, 5.32) |
| 24 | 4.76 | 0.30 | (4.18, 5.34) |
| 25 | 4.78 | 0.30 | (4.20, 5.36) |
| 26 | 4.80 | 0.30 | (4.22, 5.38) |
| 27 | 4.83 | 0.30 | (4.25, 5.41) |
| 28 | 4.88 | 0.30 | (4.30, 5.45) |
| 29 | 4.93 | 0.29 | (4.35, 5.51) |
| 30 | 5.00 | 0.29 | (4.43, 5.58) |
| 31 | 5.09 | 0.29 | (4.52, 5.66) |
| 32 | 5.20 | 0.29 | (4.63, 5.77) |
| 33 | 5.32 | 0.29 | (4.75, 5.89) |
| 34 | 5.46 | 0.29 | (4.89, 6.02) |
| 35 | 5.61 | 0.29 | (5.05, 6.17) |
| 36 | 5.78 | 0.29 | (5.22, 6.34) |
| 37 | 5.96 | 0.28 | (5.40, 6.52) |
| 38 | 6.16 | 0.28 | (5.60, 6.71) |
| 39 | 6.36 | 0.28 | (5.81, 6.92) |
| 40 | 6.59 | 0.28 | (6.04, 7.13) |

**Table S25.** Weekly effect estimates under the 4-week lag model for fractional fetal arm volume from IPCW sensitivity analysis; NICHD Fetal Growth Studies - Singletons and Fetal 3D Study.

|  | Fractional Fetal Arm Volume (AVol) | | |
| --- | --- | --- | --- |
| Gestational Age, week | Estimate | Standard Error | 95% Confidence Interval |
| 15 | 4.92 | 0.29 | (4.35, 5.49) |
| 16 | 4.91 | 0.29 | (4.34, 5.48) |
| 17 | 4.90 | 0.29 | (4.33, 5.47) |
| 18 | 4.89 | 0.29 | (4.32, 5.46) |
| 19 | 4.89 | 0.29 | (4.32, 5.46) |
| 20 | 4.90 | 0.29 | (4.33, 5.47) |
| 21 | 4.90 | 0.29 | (4.33, 5.48) |
| 22 | 4.92 | 0.29 | (4.34, 5.49) |
| 23 | 4.93 | 0.29 | (4.36, 5.51) |
| 24 | 4.95 | 0.30 | (4.37, 5.53) |
| 25 | 4.97 | 0.30 | (4.39, 5.55) |
| 26 | 5.00 | 0.30 | (4.42, 5.58) |
| 27 | 5.03 | 0.30 | (4.45, 5.61) |
| 28 | 5.07 | 0.30 | (4.49, 5.65) |
| 29 | 5.13 | 0.30 | (4.55, 5.71) |
| 30 | 5.20 | 0.30 | (4.62, 5.78) |
| 31 | 5.29 | 0.29 | (4.71, 5.86) |
| 32 | 5.39 | 0.29 | (4.82, 5.97) |
| 33 | 5.51 | 0.29 | (4.94, 6.09) |
| 34 | 5.65 | 0.29 | (5.08, 6.22) |
| 35 | 5.81 | 0.29 | (5.24, 6.37) |
| 36 | 5.97 | 0.29 | (5.41, 6.54) |
| 37 | 6.15 | 0.29 | (5.59, 6.71) |
| 38 | 6.35 | 0.28 | (5.79, 6.90) |
| 39 | 6.55 | 0.28 | (6.00, 7.11) |
| 40 | 6.77 | 0.28 | (6.22, 7.32) |

**Table S26.** Weekly effect estimates under the 5-week lag model for fractional fetal arm volume from IPCW sensitivity analysis; NICHD Fetal Growth Studies - Singletons and Fetal 3D Study.

|  | Fractional Fetal Arm Volume (AVol) | | |
| --- | --- | --- | --- |
| Gestational Age, week | Estimate | Standard Error | 95% Confidence Interval |
| 15 | 5.11 | 0.29 | (4.53, 5.69) |
| 16 | 5.09 | 0.29 | (4.52, 5.67) |
| 17 | 5.08 | 0.29 | (4.50, 5.65) |
| 18 | 5.07 | 0.29 | (4.50, 5.64) |
| 19 | 5.06 | 0.29 | (4.49, 5.63) |
| 20 | 5.06 | 0.29 | (4.49, 5.63) |
| 21 | 5.07 | 0.29 | (4.50, 5.64) |
| 22 | 5.08 | 0.29 | (4.51, 5.66) |
| 23 | 5.10 | 0.29 | (4.52, 5.68) |
| 24 | 5.12 | 0.30 | (4.54, 5.70) |
| 25 | 5.15 | 0.30 | (4.56, 5.73) |
| 26 | 5.17 | 0.30 | (4.59, 5.76) |
| 27 | 5.21 | 0.30 | (4.63, 5.79) |
| 28 | 5.25 | 0.30 | (4.67, 5.84) |
| 29 | 5.31 | 0.30 | (4.72, 5.89) |
| 30 | 5.38 | 0.30 | (4.80, 5.96) |
| 31 | 5.46 | 0.30 | (4.88, 6.04) |
| 32 | 5.57 | 0.29 | (4.99, 6.15) |
| 33 | 5.69 | 0.29 | (5.12, 6.27) |
| 34 | 5.83 | 0.29 | (5.26, 6.41) |
| 35 | 5.99 | 0.29 | (5.42, 6.56) |
| 36 | 6.15 | 0.29 | (5.59, 6.72) |
| 37 | 6.33 | 0.29 | (5.77, 6.90) |
| 38 | 6.52 | 0.29 | (5.96, 7.09) |
| 39 | 6.73 | 0.29 | (6.17, 7.29) |
| 40 | 6.94 | 0.28 | (6.39, 7.50) |

**Table S27.** Weekly effect estimates under the 6-week lag model for fractional fetal arm volume from IPCW sensitivity analysis; NICHD Fetal Growth Studies - Singletons and Fetal 3D Study.

|  | Fractional Fetal Arm Volume (AVol) | | |
| --- | --- | --- | --- |
| Gestational Age, week | Estimate | Standard Error | 95% Confidence Interval |
| 15 | 5.27 | 0.30 | (4.69, 5.86) |
| 16 | 5.25 | 0.30 | (4.67, 5.83) |
| 17 | 5.23 | 0.29 | (4.65, 5.80) |
| 18 | 5.21 | 0.29 | (4.64, 5.79) |
| 19 | 5.20 | 0.29 | (4.63, 5.78) |
| 20 | 5.20 | 0.29 | (4.63, 5.77) |
| 21 | 5.20 | 0.29 | (4.63, 5.78) |
| 22 | 5.21 | 0.29 | (4.64, 5.79) |
| 23 | 5.23 | 0.29 | (4.65, 5.81) |
| 24 | 5.25 | 0.30 | (4.67, 5.83) |
| 25 | 5.28 | 0.30 | (4.70, 5.86) |
| 26 | 5.31 | 0.30 | (4.73, 5.89) |
| 27 | 5.35 | 0.30 | (4.76, 5.93) |
| 28 | 5.39 | 0.30 | (4.81, 5.98) |
| 29 | 5.45 | 0.30 | (4.86, 6.04) |
| 30 | 5.52 | 0.30 | (4.94, 6.11) |
| 31 | 5.61 | 0.30 | (5.02, 6.19) |
| 32 | 5.71 | 0.30 | (5.13, 6.30) |
| 33 | 5.84 | 0.30 | (5.26, 6.42) |
| 34 | 5.98 | 0.29 | (5.40, 6.56) |
| 35 | 6.13 | 0.29 | (5.56, 6.71) |
| 36 | 6.30 | 0.29 | (5.73, 6.87) |
| 37 | 6.48 | 0.29 | (5.91, 7.05) |
| 38 | 6.67 | 0.29 | (6.11, 7.24) |
| 39 | 6.87 | 0.29 | (6.31, 7.44) |
| 40 | 7.09 | 0.29 | (6.53, 7.65) |

**Table S28.** Weekly effect estimates under the 7-week lag model for fractional fetal arm volume from IPCW sensitivity analysis; NICHD Fetal Growth Studies - Singletons and Fetal 3D Study.

|  | Fractional Fetal Arm Volume (AVol) | | |
| --- | --- | --- | --- |
| Gestational Age, week | Estimate | Standard Error | 95% Confidence Interval |
| 15 | 5.38 | 0.30 | (4.78, 5.97) |
| 16 | 5.35 | 0.30 | (4.76, 5.94) |
| 17 | 5.33 | 0.30 | (4.74, 5.91) |
| 18 | 5.31 | 0.30 | (4.72, 5.89) |
| 19 | 5.29 | 0.30 | (4.71, 5.87) |
| 20 | 5.28 | 0.29 | (4.71, 5.86) |
| 21 | 5.28 | 0.29 | (4.71, 5.86) |
| 22 | 5.29 | 0.29 | (4.71, 5.87) |
| 23 | 5.30 | 0.30 | (4.72, 5.88) |
| 24 | 5.32 | 0.30 | (4.74, 5.90) |
| 25 | 5.35 | 0.30 | (4.77, 5.93) |
| 26 | 5.38 | 0.30 | (4.80, 5.97) |
| 27 | 5.42 | 0.30 | (4.84, 6.01) |
| 28 | 5.47 | 0.30 | (4.88, 6.06) |
| 29 | 5.53 | 0.30 | (4.94, 6.12) |
| 30 | 5.60 | 0.30 | (5.01, 6.19) |
| 31 | 5.69 | 0.30 | (5.10, 6.28) |
| 32 | 5.80 | 0.30 | (5.21, 6.38) |
| 33 | 5.92 | 0.30 | (5.33, 6.51) |
| 34 | 6.06 | 0.30 | (5.48, 6.65) |
| 35 | 6.22 | 0.30 | (5.64, 6.80) |
| 36 | 6.39 | 0.30 | (5.81, 6.97) |
| 37 | 6.57 | 0.29 | (6.00, 7.15) |
| 38 | 6.77 | 0.29 | (6.19, 7.34) |
| 39 | 6.97 | 0.29 | (6.40, 7.54) |
| 40 | 7.18 | 0.29 | (6.61, 7.75) |

**Table S29.** Weekly effect estimates under the 8-week lag model for fractional fetal arm volume from IPCW sensitivity analysis; NICHD Fetal Growth Studies - Singletons and Fetal 3D Study.

|  | Fractional Fetal Arm Volume (AVol) | | |
| --- | --- | --- | --- |
| Gestational Age, week | Estimate | Standard Error | 95% Confidence Interval |
| 15 | 5.39 | 0.31 | (4.79, 6.00) |
| 16 | 5.36 | 0.31 | (4.76, 5.96) |
| 17 | 5.34 | 0.30 | (4.74, 5.93) |
| 18 | 5.31 | 0.30 | (4.73, 5.90) |
| 19 | 5.30 | 0.30 | (4.71, 5.88) |
| 20 | 5.28 | 0.30 | (4.70, 5.86) |
| 21 | 5.28 | 0.30 | (4.70, 5.86) |
| 22 | 5.28 | 0.30 | (4.70, 5.86) |
| 23 | 5.29 | 0.30 | (4.71, 5.87) |
| 24 | 5.30 | 0.30 | (4.72, 5.89) |
| 25 | 5.33 | 0.30 | (4.74, 5.91) |
| 26 | 5.36 | 0.30 | (4.77, 5.95) |
| 27 | 5.40 | 0.30 | (4.81, 5.99) |
| 28 | 5.45 | 0.30 | (4.86, 6.04) |
| 29 | 5.51 | 0.30 | (4.92, 6.10) |
| 30 | 5.59 | 0.30 | (4.99, 6.18) |
| 31 | 5.68 | 0.30 | (5.08, 6.27) |
| 32 | 5.78 | 0.30 | (5.19, 6.37) |
| 33 | 5.91 | 0.30 | (5.32, 6.50) |
| 34 | 6.05 | 0.30 | (5.46, 6.64) |
| 35 | 6.21 | 0.30 | (5.62, 6.80) |
| 36 | 6.38 | 0.30 | (5.80, 6.97) |
| 37 | 6.57 | 0.30 | (5.99, 7.15) |
| 38 | 6.77 | 0.30 | (6.19, 7.35) |
| 39 | 6.97 | 0.29 | (6.40, 7.55) |
| 40 | 7.18 | 0.29 | (6.61, 7.76) |

**Table S30.** Weekly effect estimates under the 9-week lag model for fractional fetal arm volume from IPCW sensitivity analysis; NICHD Fetal Growth Studies - Singletons and Fetal 3D Study.

|  | Fractional Fetal Arm Volume (AVol) | | |
| --- | --- | --- | --- |
| Gestational Age, week | Estimate | Standard Error | 95% Confidence Interval |
| 15 | 5.27 | 0.32 | (4.65, 5.89) |
| 16 | 5.24 | 0.31 | (4.63, 5.85) |
| 17 | 5.22 | 0.31 | (4.61, 5.82) |
| 18 | 5.19 | 0.30 | (4.60, 5.79) |
| 19 | 5.17 | 0.30 | (4.58, 5.76) |
| 20 | 5.16 | 0.30 | (4.57, 5.74) |
| 21 | 5.14 | 0.30 | (4.56, 5.73) |
| 22 | 5.14 | 0.30 | (4.56, 5.73) |
| 23 | 5.14 | 0.30 | (4.56, 5.73) |
| 24 | 5.16 | 0.30 | (4.57, 5.74) |
| 25 | 5.18 | 0.30 | (4.59, 5.76) |
| 26 | 5.21 | 0.30 | (4.62, 5.79) |
| 27 | 5.25 | 0.30 | (4.66, 5.84) |
| 28 | 5.30 | 0.30 | (4.70, 5.89) |
| 29 | 5.36 | 0.30 | (4.76, 5.95) |
| 30 | 5.43 | 0.30 | (4.84, 6.03) |
| 31 | 5.52 | 0.30 | (4.93, 6.12) |
| 32 | 5.63 | 0.30 | (5.03, 6.23) |
| 33 | 5.76 | 0.30 | (5.16, 6.35) |
| 34 | 5.90 | 0.30 | (5.31, 6.50) |
| 35 | 6.06 | 0.30 | (5.47, 6.66) |
| 36 | 6.24 | 0.30 | (5.65, 6.83) |
| 37 | 6.43 | 0.30 | (5.85, 7.02) |
| 38 | 6.64 | 0.30 | (6.05, 7.22) |
| 39 | 6.84 | 0.30 | (6.26, 7.43) |
| 40 | 7.06 | 0.30 | (6.48, 7.64) |

**Table S31.** Weekly effect estimates under the 10-week lag model for fractional fetal arm volume from IPCW sensitivity analysis; NICHD Fetal Growth Studies - Singletons and Fetal 3D Study.

|  | Fractional Fetal Arm Volume (AVol) | | |
| --- | --- | --- | --- |
| Gestational Age, week | Estimate | Standard Error | 95% Confidence Interval |
| 15 | 4.95 | 0.32 | (4.32, 5.58) |
| 16 | 4.94 | 0.32 | (4.32, 5.56) |
| 17 | 4.92 | 0.31 | (4.31, 5.53) |
| 18 | 4.90 | 0.31 | (4.29, 5.50) |
| 19 | 4.88 | 0.31 | (4.28, 5.48) |
| 20 | 4.86 | 0.30 | (4.27, 5.45) |
| 21 | 4.85 | 0.30 | (4.26, 5.44) |
| 22 | 4.84 | 0.30 | (4.25, 5.43) |
| 23 | 4.84 | 0.30 | (4.25, 5.43) |
| 24 | 4.85 | 0.30 | (4.26, 5.43) |
| 25 | 4.86 | 0.30 | (4.27, 5.45) |
| 26 | 4.89 | 0.30 | (4.30, 5.47) |
| 27 | 4.92 | 0.30 | (4.33, 5.51) |
| 28 | 4.97 | 0.30 | (4.38, 5.56) |
| 29 | 5.03 | 0.30 | (4.43, 5.62) |
| 30 | 5.10 | 0.30 | (4.50, 5.70) |
| 31 | 5.19 | 0.30 | (4.59, 5.79) |
| 32 | 5.30 | 0.31 | (4.70, 5.90) |
| 33 | 5.43 | 0.31 | (4.83, 6.03) |
| 34 | 5.58 | 0.30 | (4.98, 6.17) |
| 35 | 5.74 | 0.30 | (5.14, 6.34) |
| 36 | 5.92 | 0.30 | (5.33, 6.52) |
| 37 | 6.12 | 0.30 | (5.53, 6.71) |
| 38 | 6.33 | 0.30 | (5.74, 6.92) |
| 39 | 6.55 | 0.30 | (5.96, 7.13) |
| 40 | 6.77 | 0.30 | (6.18, 7.35) |

**Table S32.** Weekly effect estimates under the 11-week lag model for fractional fetal arm volume from IPCW sensitivity analysis; NICHD Fetal Growth Studies - Singletons and Fetal 3D Study.

|  | Fractional Fetal Arm Volume (AVol) | | |
| --- | --- | --- | --- |
| Gestational Age, week | Estimate | Standard Error | 95% Confidence Interval |
| 15 | 4.41 | 0.33 | (3.77, 5.05) |
| 16 | 4.41 | 0.32 | (3.78, 5.04) |
| 17 | 4.41 | 0.32 | (3.79, 5.03) |
| 18 | 4.40 | 0.31 | (3.78, 5.01) |
| 19 | 4.38 | 0.31 | (3.78, 4.99) |
| 20 | 4.37 | 0.31 | (3.77, 4.97) |
| 21 | 4.36 | 0.30 | (3.76, 4.95) |
| 22 | 4.35 | 0.30 | (3.76, 4.94) |
| 23 | 4.35 | 0.30 | (3.76, 4.93) |
| 24 | 4.35 | 0.30 | (3.76, 4.93) |
| 25 | 4.36 | 0.30 | (3.77, 4.94) |
| 26 | 4.38 | 0.30 | (3.79, 4.96) |
| 27 | 4.41 | 0.30 | (3.82, 4.99) |
| 28 | 4.45 | 0.30 | (3.86, 5.04) |
| 29 | 4.50 | 0.30 | (3.91, 5.09) |
| 30 | 4.57 | 0.30 | (3.98, 5.17) |
| 31 | 4.66 | 0.30 | (4.06, 5.26) |
| 32 | 4.77 | 0.30 | (4.17, 5.37) |
| 33 | 4.90 | 0.31 | (4.30, 5.49) |
| 34 | 5.04 | 0.31 | (4.45, 5.64) |
| 35 | 5.21 | 0.30 | (4.62, 5.81) |
| 36 | 5.40 | 0.30 | (4.80, 6.00) |
| 37 | 5.60 | 0.30 | (5.01, 6.20) |
| 38 | 5.82 | 0.30 | (5.23, 6.41) |
| 39 | 6.04 | 0.30 | (5.46, 6.63) |
| 40 | 6.27 | 0.30 | (5.69, 6.86) |

**Table S33.** Weekly effect estimates under the 12-week lag model for fractional fetal arm volume from IPCW sensitivity analysis; NICHD Fetal Growth Studies - Singletons and Fetal 3D Study.

|  | Fractional Fetal Arm Volume (AVol) | | |
| --- | --- | --- | --- |
| Gestational Age, week | Estimate | Standard Error | 95% Confidence Interval |
| 15 | 3.63 | 0.33 | (2.98, 4.28) |
| 16 | 3.66 | 0.33 | (3.02, 4.30) |
| 17 | 3.68 | 0.32 | (3.05, 4.30) |
| 18 | 3.68 | 0.32 | (3.06, 4.30) |
| 19 | 3.68 | 0.31 | (3.07, 4.29) |
| 20 | 3.68 | 0.31 | (3.07, 4.28) |
| 21 | 3.67 | 0.30 | (3.07, 4.27) |
| 22 | 3.67 | 0.30 | (3.07, 4.26) |
| 23 | 3.66 | 0.30 | (3.08, 4.25) |
| 24 | 3.67 | 0.30 | (3.08, 4.25) |
| 25 | 3.67 | 0.30 | (3.09, 4.26) |
| 26 | 3.69 | 0.30 | (3.10, 4.27) |
| 27 | 3.71 | 0.30 | (3.13, 4.30) |
| 28 | 3.75 | 0.30 | (3.16, 4.33) |
| 29 | 3.79 | 0.30 | (3.21, 4.38) |
| 30 | 3.86 | 0.30 | (3.27, 4.45) |
| 31 | 3.94 | 0.30 | (3.35, 4.53) |
| 32 | 4.04 | 0.30 | (3.45, 4.64) |
| 33 | 4.17 | 0.30 | (3.57, 4.76) |
| 34 | 4.32 | 0.30 | (3.72, 4.91) |
| 35 | 4.49 | 0.30 | (3.89, 5.08) |
| 36 | 4.67 | 0.30 | (4.08, 5.27) |
| 37 | 4.88 | 0.30 | (4.29, 5.48) |
| 38 | 5.11 | 0.30 | (4.52, 5.70) |
| 39 | 5.34 | 0.30 | (4.75, 5.93) |
| 40 | 5.58 | 0.30 | (5.00, 6.17) |

**Table S34.** Weekly effect estimates under the 13-week lag model for fractional fetal arm volume from IPCW sensitivity analysis; NICHD Fetal Growth Studies - Singletons and Fetal 3D Study.

|  | Fractional Fetal Arm Volume (AVol) | | |
| --- | --- | --- | --- |
| Gestational Age, week | Estimate | Standard Error | 95% Confidence Interval |
| 15 | 2.64 | 0.33 | (1.99, 3.29) |
| 16 | 2.70 | 0.33 | (2.06, 3.34) |
| 17 | 2.75 | 0.32 | (2.11, 3.38) |
| 18 | 2.78 | 0.32 | (2.15, 3.40) |
| 19 | 2.80 | 0.31 | (2.18, 3.41) |
| 20 | 2.81 | 0.31 | (2.20, 3.41) |
| 21 | 2.81 | 0.30 | (2.22, 3.41) |
| 22 | 2.82 | 0.30 | (2.23, 3.41) |
| 23 | 2.82 | 0.30 | (2.24, 3.41) |
| 24 | 2.83 | 0.30 | (2.24, 3.41) |
| 25 | 2.84 | 0.30 | (2.26, 3.42) |
| 26 | 2.85 | 0.30 | (2.27, 3.43) |
| 27 | 2.87 | 0.30 | (2.29, 3.45) |
| 28 | 2.90 | 0.30 | (2.32, 3.48) |
| 29 | 2.94 | 0.30 | (2.36, 3.52) |
| 30 | 2.99 | 0.30 | (2.41, 3.58) |
| 31 | 3.07 | 0.30 | (2.48, 3.65) |
| 32 | 3.16 | 0.30 | (2.58, 3.75) |
| 33 | 3.28 | 0.30 | (2.69, 3.87) |
| 34 | 3.43 | 0.30 | (2.84, 4.02) |
| 35 | 3.59 | 0.30 | (3.00, 4.18) |
| 36 | 3.78 | 0.30 | (3.19, 4.37) |
| 37 | 3.99 | 0.30 | (3.40, 4.58) |
| 38 | 4.22 | 0.30 | (3.64, 4.81) |
| 39 | 4.47 | 0.30 | (3.88, 5.05) |
| 40 | 4.72 | 0.30 | (4.14, 5.31) |

**Table S35.** Weekly effect estimates under the 14-week lag model for fractional fetal arm volume from IPCW sensitivity analysis; NICHD Fetal Growth Studies - Singletons and Fetal 3D Study.
